# Previously Uncharacterised Aliphatic Amino Acid Positions Modulate the Apparent Catalytic Activity of the EAL Domain of ZMO_1055 and Other Cyclic Di‐GMP‐Specific EAL Phosphodiesterases

**DOI:** 10.1111/1751-7915.70308

**Published:** 2026-02-12

**Authors:** Lian‐Ying Cao, Xue Zhang, Feng‐wu Bai, Ute Römling

**Affiliations:** ^1^ Department of Microbiology, Tumor and Cell Biology, Biomedicum Karolinska Institutet Stockholm Sweden; ^2^ State Key Laboratory of Microbial Metabolism, Joint International Research Laboratory of Metabolic & Developmental Science, and School of Life Science and Biotechnology Shanghai Jiao Tong University Shanghai China

## Abstract

The nearly ubiquitous second messenger cyclic di‐GMP signalling system decides about the bacterial lifestyle transition between sessility and motility. GGDEF diguanylate cyclase and EAL phosphodiesterase domains conventionally conduct the turnover of the signalling molecule being subject to micro‐ and macroevolution. While highly conserved signature amino acids involved in divalent cation binding and catalysis have readily been identified, recognition of single amino acid substitutions that modulate the catalytic activity has been rare. Associated with development towards cellulose‐mediated self‐flocculation in *Zymomonas mobilis* ZM401, the A526V substitution and substitutions with amino acids with longer aliphatic side chains gradually revert the apparent catalytic activity of the EAL domain in the PAS‐GGDEF‐EAL ZMO1055 phosphodiesterase as monitored by flocculation, biofilm and motility assays. Remarkably, the effect of the A526V substitution equivalent is observed among other investigated GGDEF‐EAL proteins. Furthermore, substitutions of aliphatic side chain amino acids at distinct alternative positions affect ZOM1055 activity, while the M525L substitution has a context‐dependent effect. Thus, single amino acid substitutions outside of signature amino acid positions can even revert the target output and thus significantly contribute to the flexibility and adaptability of the cyclic di‐GMP signalling network. At a phylogenetic scale, ZMO1055 homologues seem to be a current evolutionary target.

## Graphical abstract

The A526V substitution, previously not recognised to affect the functionality of the EAL domain, downregulates the apparent catalytic activity of the PAS‐GGDEF‐EAL ZMO1055 phosphodiesterase and other GGDEF‐EAL proteins. Substitution of A526, which is conserved among homologues, with amino acids with longer aliphatic side chains than valine, reverts to an apparent diguanylate cyclase activity.

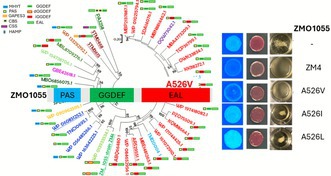

## 1 Introduction

To date, a vast number of cyclic nucleotide‐based second messenger molecules have been identified in bacteria (Ross et al. 1987; Whiteley et al. 2019; Witte et al. 2008). Remarkably, prior to discovery in vivo, some of these molecules had already been chemically synthesised earlier and were predicted by in vitro experimentation to possess biological activity (de Vroom et al. 1988; Hsu and Dennis 1982; Ross et al. 1990). However, among these compounds, cyclic di‐GMP seems to be (one of) the most widespread bona fide second messenger molecules (Römling 2023; Römling et al. 2013). Synthesised by members of the GGDEF domain superfamily and hydrolysed by members of the EAL domain superfamily, the complexity of the cyclic di‐GMP network is reflected by its regular occurrence in genomes throughout the phylogenetic tree, the multiplicity of cyclic di‐GMP turnover proteins encoded by one bacterial genome and the structural diversity of receptors harboured by an individual bacterial genome (Römling et al. 2005; Schirmer and Jenal 2009). As in all domain superfamilies, members of the GGDEF and EAL superfamilies are subject to macro‐ and microevolution (Beyhan and Yildiz 2007; Römling et al. 2023) where evolution towards catalytically inactive and/or substrate or product binding receptor domains is regularly identified. Intriguingly, ongoing microevolution, may it be in the laboratory setting or observed in closely related isolates or species, allows identification of evolutionary forces and evolutionary mechanisms leading to the initiation of alterations in the amino acid sequence in almost real time (Cimdins et al. 2017; Farr et al. 2017; Sun et al. 2014; Zlatkov and Uhlin 2019). For example, originally identified as a catalytically incompetent protein and characterised as a small RNA binding protein in commensal *Escherichia coli* , the GAPES4‐HAMP‐GGDEF‐EAL protein CsrD can exhibit cyclic di‐GMP binding and even catalytic activity in other species of the Enterobacteriaceae (Fineran et al. 2007; Kharadi and Sundin 2022; Potts et al. 2018). Equally, the three most closely related EAL domain proteins encoded by the *Salmonella typhimurium* genome either display catalytic activity or work exclusively through protein–protein interactions (Ahmad et al. 2013; El Mouali et al. 2017; Simm et al. 2009).

*Zymomonas mobilis* is an organism with a high potential to be applied in industry for lignocellulosic ethanol production (Xia et al. 2019). In this context, selection of the *Z. mobilis* ZM4 strain for enhanced ethanol tolerance by nitrosoguanidine‐based mutagenesis created the self‐flocculating strain *Z. mobilis* ZM401 (Lee et al. 1982; Zhao et al. 2014). Sequencing of *Z. mobilis* ZMO401 identified 32 single nucleotide polymorphisms and one single nucleotide deletion in a poly‐nucleotide tract (Cao et al. 2022; Jeon et al. 2012; Zhao et al. 2014). Thereby, two amino acid alterations were considered most relevant for the self‐flocculating phenotype. The single nucleotide deletion that led to the fusion of the ZMO1082 and ZMO1083 genes resulted in a longer BcsA cellulose synthase open reading frame and gene product. On the other hand, the nonsynonymous single nucleotide polymorphism C1577T created the A526V amino acid substitution in the EAL domain of the GGDEF‐EAL protein ZMO1055_ZM4_, subsequently designated ZMO1055_ZM401_, to display a lower apparent phosphodiesterase activity and reduced capacity to dissolve the self‐flocculation phenotype caused by elevated cellulose production in strain *Z. mobilis* ZMO401 (Cao et al. 2022; Jeon et al. 2012). Indeed, deletion and overexpression analysis in combination with mutant construction of all cyclic di‐GMP turnover proteins in *Z. mobilis* ZM4 and ZM401 showed that ZMO1055 is a key diguanylate cyclase/phosphodiesterase regulating cellulose‐triggered self‐flocculation (Li, Xia, et al. 2023).

Recognising the impact of the A526V substitution located in an amino acid stretch of the EAL domain not previously identified as functionally relevant, we were asking whether the impact of the A526V substitution is observed in other model systems, whether the amino acid position equivalent to A526 in ZMO1055 is conserved among the EAL domains and whether functional conservation of the A526V substitution occurs. We found that the consequence of the A526V substitution of ZMO1055 is conserved in functional model systems in unrelated organisms. Alanine is predominant at the 526 position equivalent in ZMO1055 homologues and other EAL domain proteins with the substitution of alanine 526 and upstream position 525 by amino acids with a more bulky aliphatic, polar, or helix‐breaking side chain to mediate alterations of apparent catalytic activity which can be functionally conserved and context dependent occurring in more than one subgroup of EAL domain proteins. However also non‐signature amino acids at other positions can alter the apparent catalytic activity of ZMO1055.

## 2 Experimental Procedures

### 2.1 Strains and Growth Conditions

The strains used in this work are listed in Table S1. *E. coli* and *S. typhimurium* strains were cultivated in Luria‐Bertani (LB: 1% tryptone, 0.5% yeast extract, 1% NaCl) medium, *Z. mobilis* ZM4 strains and derivatives were cultivated in Rich‐Medium (RM, 1% yeast extract, 2% glucose, 0.2% KH_2_PO_4_) starting with an initial OD_600_ = 0.1. Strains containing the pEZ plasmid were cultivated in medium supplied with 100 mg/L spectinomycin, strains harbouring the pBAD28 or pBAD30 plasmids were supplied with 25 mg/L chloramphenicol and 100 mg/L ampicillin, respectively. 100 ng/L anhydrotetracycline and 0.1% L‐arabinose were applied to induce gene expression from the pEZ and pBAD plasmids, respectively.

### 2.2 Plasmid Construction

The pEZ backbone was constructed by Golden Gate cloning (Engler et al. 2008). The plasmid backbone and the P_aTc_ promoter were amplified from plasmid pEZ‐dual promoter (Yang et al. 2019). Genes *ZMO1055*_*ZM4*_ and *ZMO1055*_*ZM401*_ were amplified from the genomes of *Z. mobilis* ZM4 and ZM401, respectively. Plasmid backbone, promoter, and gene fragments were purified by Cycle Pure (Omega), restricted with BsaI (NEB Biolabs), and then ligated with T4 DNA ligase (NEB Biolabs) using the Golden Gate cloning protocol (Engler et al. 2008). Plasmids with gene fragments were assembled by restriction site‐mediated ligation and alternatively by in vivo cloning (Bubeck et al. 1993; Jones and Howard 1991). Recombinant plasmids or linear fragments were transformed into *E. coli* K‐12 TOP10 to be confirmed by PCR and sequencing. Verified plasmids were transformed into *Z. mobilis* ZM401 and *S. typhimurium* UMR1 Δ*yhjH* for the assessment of self‐flocculation, biofilm morphotypes and motility. Plasmids used and constructed in this work are provided in Table S2. Primers can be found in Table S3.

### 2.3 Site‐Directed Mutagenesis

Site‐directed mutagenesis was performed by the Q5 site‐directed mutagenesis kit (NEB Biolabs) with primers designed to harbour the mutation site(s). After transformation into the *E. coli* K‐12 host, the mutated open reading frames were amplified by PCR and confirmed by Sanger sequencing. Verified plasmids were transformed by electroporation (Biorad Gene Pulser) into their respective *Z. mobilis* ZM401 and *S. typhimurium* UMR1 Δ*yhjH* hosts for in vivo assessment of protein function.

### 2.4 Congo Red Binding Assay for *Z. mobilis*

A Congo red binding assay was set up to detect cyclic di‐GMP mediated alterations in colony morphology including biosynthesis of cellulose of *Z. mobilis* ZM401 on agar plates. Shortly, a loopful of bacteria was collected from a RM agar plate and inoculated into Rich‐medium containing the appropriate antibiotic with initial OD_600_ = 0.1. After cultivating at 30°C for 12 h, the bacteria were collected and re‐suspended in RM and subsequently adjusted to OD_600_ = 5.0 as seed culture. Three μl of seed culture were carefully positioned onto a RM‐medium‐based Congo red plate (2% glucose, 1% yeast extract, 0.2% KH_2_PO_4_, 40 μg/mL Congo red, 20 μg/mL Coomassie Brilliant Blue (CBB) G‐250, 1.5% agar), the spot dried and incubated at 30°C. The Congo red morphotype was documented after 24 and 48 h.

### 2.5 Calcofluor White Binding Assay to Assess Cellulose Production of *Z. mobilis*

Calcofluor white (fluorescent brightener 28) binding of bacterial colonies was monitored to assess the expression of cellulose. A seed culture was prepared as described for the Congo red assay. Three μl of seed culture were carefully placed onto a Calcofluor white agar plate (2% glucose, 1% yeast extract, 0.2% KH_2_PO_4_, 50 μg/mL Calcofluor white, 1.5% agar). After drying of the spot, the plate was incubated at 30°C. The calcofluor white binding was documented after 24 h upon exposure to UV light of wavelength 365 nm.

### 2.6 *S. typhimurium* Swimming Assay

Swimming motility of *S. typhimurium* UMR1 Δ*yhjH* was quantified by the diameter of the swimming halo (Li et al. 2019). Shortly, a single colony was picked and streaked onto an LB medium plate containing the suitable antibiotic. After incubation of the plate at 30°C overnight, the bacteria were re‐suspended in LB without NaCl medium and subsequently adjusted to OD_600_ = 5.0 as seed culture. Seed culture of 3 μL were injected into soft agar LB medium (1% tryptone, 0.5% yeast extract, 1% NaCl, 0.25% agar) and the plate incubated at 30°C. Recovery of enhanced swimming motility of *S. typhimurium* UMR1 Δ*yhjH* served as a proxy for the degree of apparent PDE activity. The swimming diameter was documented each hour from 5 to 9 h.

### 2.7 Rdar Colony Morphotype Assay for *S. typhimurium*

The rdar morphotype to reflect the cyclic di‐GMP dependent expression of the extracellular matrix components cellulose and curli fimbriae via the rdar biofilm activator CsgD was visualised as follows: A seed culture was prepared as described for the swimming motility assay. Three μl of seed culture were carefully placed onto a Congo red agar plate (1% tryptone, 0.5% yeast extract, 40 μg/mL Congo red, 20 μg/mL CBB G‐250, 1.5% agar). After drying of the spot, the plate was incubated at 30°C. Alterations in colony morphology and dye uptake, conventionally designated as rdar morphotype, were documented after 24 h.

### 2.8 Western Blot Analysis

To detect protein production, 5 mg of *Z. mobilis* cells were collected from a liquid culture by centrifugation, resuspended in 200 μL sodium dodecylsulfate (SDS) sample buffer and heated at 95°C for 10 min. For *S. typhimurium* strains, 5 mg of cells were collected and re‐suspended in 100 μL SDS sample buffer. The protein concentration was assessed by CBB G‐250 staining after running of the protein extracts on an SDS‐PAGE (4% stacking and 12% resolving gel). Samples normalised for the protein content were separated by SDS‐PAGE and transferred onto a polyvinylidene fluoride (PVDF) membrane (Millipore). After washing with TBS buffer twice, the membrane was blocked with Anti·His horseradish peroxidase (HRP) Conjugate blocking buffer (Penta·His HRP Conjugate, Qiagen) at 4°C overnight. The following steps were conducted at the manufacturer's instructions with 1:2000 dilution of the HRP‐coupled anti‐His‐tag antibody (Qiagen). With the ECL light detection reagent (Roche) as substrate, chemoluminescence was detected using the Luminescent Image Analyser (LAS‐1000plus; Fujifilm).

### 2.9 Bioinformatic Analyses Including Protein Alignment and Construction of the Phylogenetic Tree

5039 non‐redundant proteins most similar to ZMO1055 as retrieved by BLAST search (April 2021) from the NCBI database (Altschul et al. 1990) and all other relevant GGDEF/EAL proteins, partially obtained from UniProt (UniProt Consortium 2018), were aligned with ZMO1055_ZM4_ by ClustalX 2.1 (Higgins and Sharp 1988) and manually curated in GeneDoc (GeneDocgd322700.exe). All phylogenetic trees (Maximum‐Likelihood with 100 or 1000 bootstrap iterations) were constructed and visualised with MEGA 7.0 or 11.0 (Kumar et al. 2016). Alternatively, visualisation of the phylogenetic relationship was done with Evolview (Zhang et al. 2012). The DGC activity of GGDEF domains was categorised in three different classes after alignment with experimentally assessed reference domains: GGDEF domains with all amino acid signatures conserved compared to catalytically competent reference GGDEF domains were classified as DGC active domains; domains with amino acids altered in functionally relevant signature motifs were classified to lack DGC activity, while minor signature alterations indicated uncertain DGC activity. For PDE activity, EAL domains with all signature amino acids conserved as compared to catalytically functional reference domains were classified as PDE active domains; domains with the catalytic base glutamine in the EGVE motif not conserved were predicted to be PDE inactive domains, and all the other domains were classified to be uncertain to possess PDE activity.

Sequence logos of aligned domains have been constructed with WebLogo 2.0 (Crooks et al. 2004; Schneider and Stephens 1990). Aligned protein sequences have been visualised with ESPript 3.0 (Robert and Gouet 2014). EasyFig (Sullivan et al. 2011) has been used to visualise alignments of sequence contigs using standard parameters. Structural models for ZMO1055 have been created with Phyre2 (Kelley et al. 2015), SWISS‐MODEL (Guex et al. 2009) or I‐Tasser (Yang et al. 2015). The Phyre2 highest scoring templates derived for the ZMO1055 GGDEF domain from PA0575, PA0861, and PA1120, and for the EAL domain from MorA (PA4601), PA0575, and BifA, all from *P. aeruginosa* . Subsequently, structural models of the wild type and variant proteins, including predicted dimers, were constructed with AlphaFold 3 (Abramson et al. 2024; Wee and Wei 2024). Structures and structural models have been visualised with Chimera 1.18 (Pettersen et al. 2004).

## 3 Results

### 3.1 Initial Characterisation of the GGDEF/EAL Domain Proteins of *Zymomonas mobilis* ZM4

The *Zymomonas mobilis* ZM4 genome codes for five GGDEF/EAL domain containing proteins. In order to associate their catalytic capability with sequence conservation, the conserved sequence motifs were analysed after domain alignment with functional DGCs/PDEs. Four proteins, ZMO0919, ZMO1365, ZMO0401, and ZMO1055 contain a GGDEF domain (Figures S1a and 1A; Li, Xia, et al. 2023). The GGDEF domains of ZMO0919 and ZMO1365 possess all signature amino acid motifs except residue leucine L29 which was substituted by isoleucine (I) and methionine (M) for ZMO0919 and ZMO1365, respectively. Leucine, isoleucine and methionine are all non‐polar amino acids and have a similar size of side chain. Substantial DGC activity of ZMO0919 and ZMO1365 has been observed as predicted (Li, Xia, et al. 2023).

**FIGURE 1:**
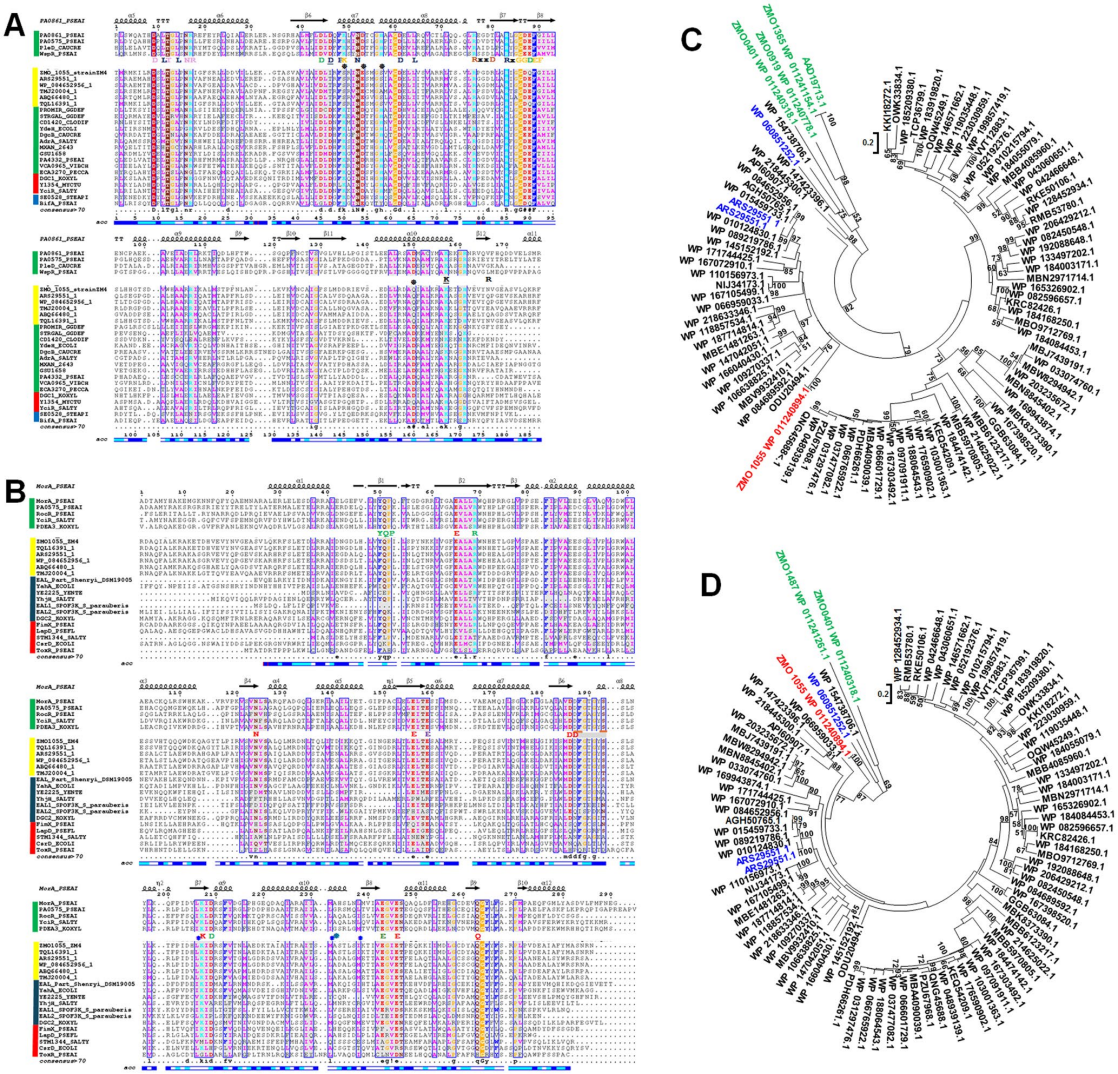
Alignment of the GGDEF and EAL domain of the diguanylate cyclase/phosphodiesterase ZMO1055_ZM4_ with the respective domains of most similar proteins and reference GGDEF and EAL domains. (A) Alignment of the ZMO1055_ZM4_ GGDEF domain with functionally characterised GGDEF domains (Liu et al. 2020) and GGDEF domains from most similar GGDEF‐EAL domain proteins (see Figure S4e; yellow bar) The GGDEF domain of the GGDEF‐EAL protein PA0861 (PDB: 5XGD) has been taken as the highest scoring template as reference for secondary structure‐sequence correlation. Functionally assigned amino acids from conserved signature motifs are indicated below the PleD ( *Caulobacter vibrioides* ) and WspR ( *Pseudomonas aeruginosa* ) reference amino acid sequences. Stars indicate amino acids not conserved in the GGDEF domain of ZMP1055 and its homologues. Functionality of amino acids in light blue, wide turn in protein; in dark blue, substrate interacting residues; in green, Mg^2+^ binding; in dark yellow, stabilising the transition state; conserved in plum, allosteric I‐site; GG[D/E]EF motif in yellow; underlined, salt bridge. Selected demonstrated catalytically functional class I GGDEF domains (indicated by green bar), catalytically functional class II GGDEF domains of GGDEF‐EAL proteins (indicated by red bar) and catalytically non‐functional class III GGDEF domains (indicated by blue bar) have been used in the alignment. (B) Alignment of ZMO1055_ZM4_ EAL domain with functionally characterised EAL domains and EAL domains from most similar GGDEF‐EAL domain proteins (see Figure S2e; yellow bar). The EAL domain of the phosphodiesterase MorA (PA4601) (PDB: 4RNJ) as highest scoring template in Phyre2 modelling has been taken as reference for secondary structure–sequence correlation. Green star indicates amino acid A526 and blue stars indicate amino acids 499, 525 and 531 substituted in the course of this work. Functionally assigned amino acids from conserved signature motifs are indicated below the PA0861 and RocR reference amino acid sequences. Functionality of amino acids in green, amino acids involved in substrate binding; in red, amino acids involved in Mg^2+^ binding; in plum, loop 6 stabilising glutamate; and in light brown, the catalytic base. Underlined in grey, loop 6; underlined in plum, mutated loop 6 amino acids. Selected experimentally confirmed functional class I EAL domains with conserved signature amino acids (green bar); functional class IIa and IIb EAL domains with partially deviating signature amino acids (red bar) and non‐functional class IIIa and IIIb EAL domains (blue bar) have been used in the alignment. (C) Phylogenetic tree of GGDEF domains of proteins most similar to ZMO1055_ZM4_. In red, ZMO1055 GGDEF domain; in green, GGDEF domains of *Z. mobilis* ZM4; in blue, GGDEF domains of proteins used for the 526 position equivalent substitution; in violet, GGDEF domain sequence displayed in the alignment in Figure 1a. Proteins used for the alignment and tree construction are indicated in Supporting Information. Proteins were aligned with ClustalX 2.1 (Higgins and Sharp 1988) and alignments subsequently manually curated in GeneDoc with the restriction to the GGDEF domain. Phylogenetic trees were created and visualised with MEGA (Kumar et al. 2016). Identical proteins domains were quality control for the alignment. (D) Phylogenetic tree of EAL domains of proteins most similar to ZMO1055_ZM4_. In red, ZMO1055 EAL domain; in green, EAL domains of *Z. mobilis* ZM4; in blue, EAL domains of proteins used for the 526 position equivalent substitution in violet, EAL domain sequence displayed in the alignment in (B). Proteins used for the alignment and tree construction are indicated in Supporting Information. Proteins were aligned with ClustalX 2.1 (Higgins and Sharp 1988) and alignments subsequently manually curated in GeneDoc with the restriction to the EAL domain. Phylogenetic trees were created and visualised with MEGA (Kumar et al. 2016). Identical protein domains were the quality control for the alignment.

The GGDEF domains of ZMO0401 and ZMO1055, however, possess a degenerated GGDEF motif altered to GNDEF and GGDQF. The substrate interacting residue aspartate D191 and the transition state stabilising residue lysine K194 are not conserved in ZMO1055_ZM4_ as in the GGDEF domains of highly similar GGDEF‐EAL domain proteins (Figure 1A). We have, however, recently shown that the GNDEF/GGDQF domains of ZMO0401 and ZMO1055 possess diguanylate cyclase catalytic activity (Li, Xia, et al. 2023).

Proteins ZMO0401, ZMO1487 and ZMO1055 contain an EAL domain which conventionally displays PDE activity. Possessing a conservative and a non‐conservative amino acid exchange in the signature motifs, tyrosine Y341 had been substituted by phenylalanine (F) in ZMO0401 and ZMO1487 and N417 substituted by methionine (M) in ZMO1487, respectively. With all other signature amino acid motifs present, including the catalytic base glutamate in the EGxE motif, PDE activity has been demonstrated for ZMO0401 and ZMO1487. The signature amino acid motifs required for catalytic activity of the EAL domain of ZMO1055 are conserved as expected from its apparent PDE activity (Figures 1B and S1b; Li, Xia, et al. 2023).

Our previous result indicated that the C1577T mutation in the ZMO1055 open reading frame resulting in the A526V substitution inhibited the apparent PDE activity (Cao et al. 2022). However, a specific function has not been assigned to the 526 position equivalent. We were therefore wondering about the impact of the A526 position in EAL domains. As a first step, the highest scoring templates for a structural EAL domain model for ZMO1055 in the Phyre2 analysis, the GGDEF‐EAL domain proteins MorA (PA4601) and PA0575 of *Pseudomonas aeruginosa* PAO1 served as reference sequences. Note that although the amino acid sequence identity is only 40.1% (similarity 56.7%), alanine at the position equivalent 526 is present in the EAL domain of the reference protein MorA, but not in PA0575.

In contrast, in the *Z. mobilis* EAL domains, alanine at the amino acid position equivalent 526 of ZMO1055 is not conserved (Figure S1b; Rao et al. 2009). Of note, the GGDEF and EAL domains of ZMO1055 are distantly related to the other GGDEF and EAL domains of the *Z. mobilis* ZM4 cyclic di‐GMP turnover proteins (Figure 1C,D). Furthermore, the GGDEF and EAL domain of ZMO1055 had not co‐evolved as distinct phylogenetic relationships are observed using proteins with GGDEF domains most similar to the ZMO1055 GGDEF domain as references (Figure 1C,D).

### 3.2 Initial Characterisation of Basic Catalytic Features of the GGDEF‐EAL Phosphodiesterase ZMO1055

Complex GGDEF‐EAL containing cyclic di‐GMP turnover proteins, although catalytically competent, can affect target output through a variety of different mechanisms. Besides diguanylate cyclase/phosphodiesterase catalytic activity, alternative catalytic activity and a scaffold function independent of the catalytic activity have been observed. Such an example is YciR, a GGDEF‐EAL domain protein of *Escherichia coli* and *Salmonella typhimurium* , which can modulate expression of the rdar biofilm activator *csgD* and subsequently rdar biofilm formation by interaction with alternative diguanylate cyclases and transcriptional regulators, and potentially alternative catalytic activity, respectively (Ahmad et al. 2017; Li, Yin, et al. 2023; Lindenberg et al. 2013).

To assess the impact of distinct amino acid substitutions on the phosphodiesterase activity of ZMO1055 in *Z. mobilis* ZM401, we first set up phenotypic assays that can sensitively reflect alterations in the catalytic activity. Of note, we designed experimental conditions where the expression of ZMO1055 and its variants will allow the assessment of either lower or higher apparent PDE activity. In this context, we first investigated whether ZMO1055 exerts its effect on cellular de‐flocculation in *Z. mobilis* ZM401 due to its phosphodiesterase activity (Li, Xia, et al. 2023) as this type of phenotypic alteration can also be caused by overexpression of a cyclic di‐GMP binding protein (Merighi et al. 2007). We chose to create alanine substitutions for E356 in the conserved EAL motif with the glutamate involved in divalent cation binding and for the catalytic base E536 in the E_536_GVE conserved motif required for catalytic activity. These mutants also served as a negative control to assess potential differences in the apparent DGC and PDE activity between ZMO1055_ZM4_ and ZMO1055_ZM401_.

**FIGURE 2:**
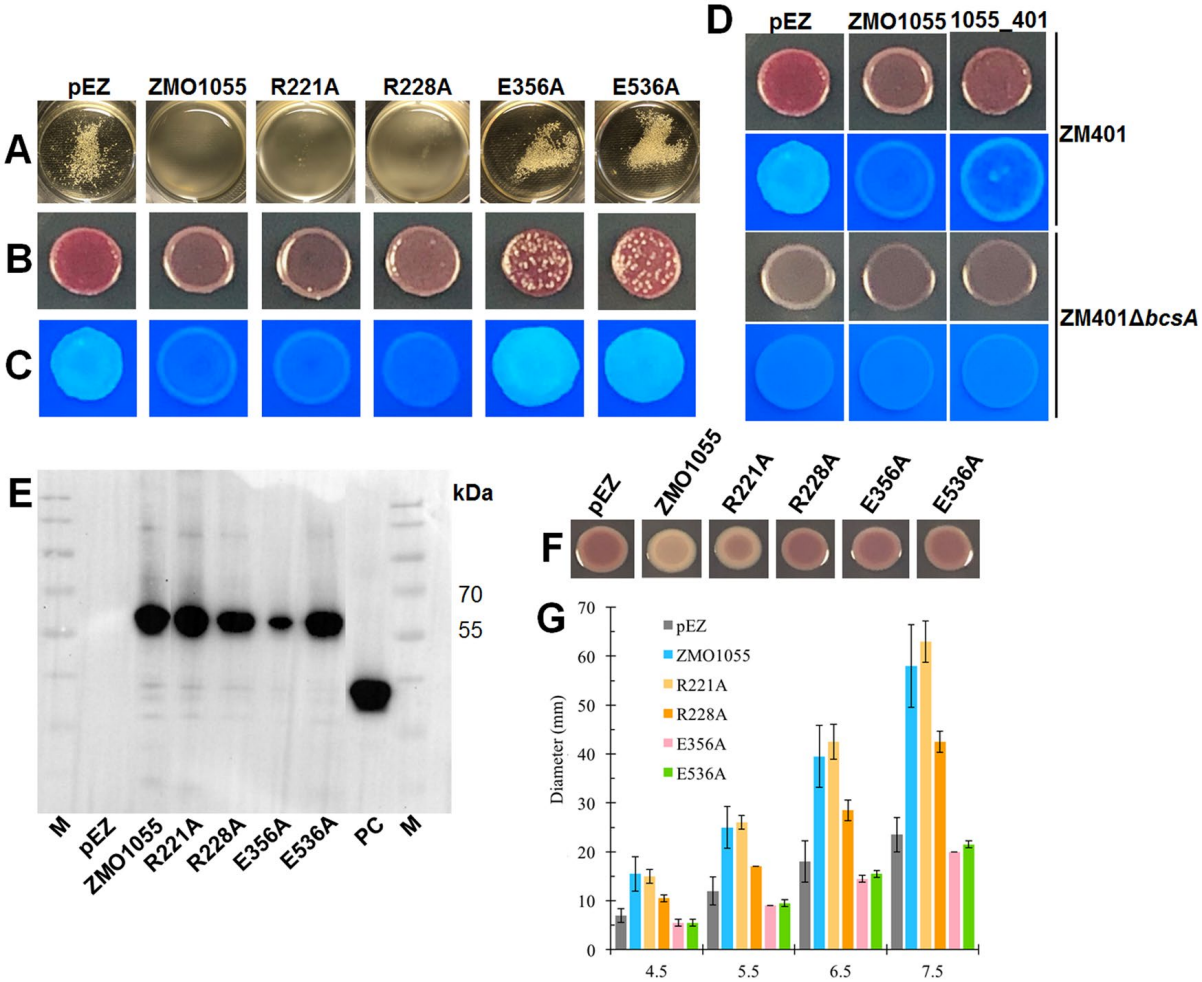
Phosphodiesterase activity of ZMO1055 from *Z. mobilis* ZM4 is required for downregulation of flocculation in *Z. mobilis* ZM401 and up‐ and downregulation of motility and biofilm formation in *S. typhimurium* UMR1 Δ*yhjH*. Flocculation in liquid medium (A), Congo red assay (B) and Calcofluor white staining assay (C) of *Z. mobilis* ZM401 over‐expressing wild‐type ZMO1055_ZM4_ or its DGC/PDE inactivated mutants after 48 and 24 h, respectively. Colony morphology morphotype of *Z. mobilis* ZM401 and *Z*. *mobilis* ZM401 Δ*bcsA* upon overexpression of ZMO1055_zm4_ and zmo1055_zm401_ in pEZ after 120 h (D). Protein expression of ZMO1055_ZM4_ and mutants (E), CapV with 6xHis‐tag from *E. coli* MG1655 (Li et al. 2022) was applied as a positive control for Western blotting. The rdar colony morphology assay (F) and swimming motility (G) of the *S. typhimurium* UMR1 Δ*yhjH* strain expressing wild‐type ZMO1055_ZM4_ or DGC/PDE inactivated mutants.

As a reference phenotype, upon expression of the GGDEF‐EAL protein ZMO1055_ZM4_ in the constitutively flocculating strain *Z. mobilis* ZM401, the self‐flocculation phenotype was completely disrupted as compared with the vector control (Figure 2; Li, Xia, et al. 2023). Expression of ZMO1055_ZM4_ EAL domain variants E356A and E536A, however, did not alter the self‐flocculation phenotype of strain *Z. mobilis* ZM401, indicating that both E356 and E536 are essential for phosphodiesterase activity of ZMO1055_ZM4_ and that the PDE activity of the EAL domain is required to dissolve self‐flocculation (Figure 2A). *Z. mobilis* ZM401 does not only show self‐flocculation, but also displays a characteristic dye binding morphotype on Congo red (Figure 2B) and Calcofluor white agar plates (Figure 2C). Mutant analysis showed that this dye binding morphotype, as the self‐flocculation, is due to expression of the exopolysaccharide cellulose (Figure 2B–D). The colony morphology and dye uptake capability was included as an alternative assessment of the catalytic activity of ZMO1055_ZM4_ and its mutants. Production of ZMO1055_ZM4_ showed downregulation of the characteristic red rdar‐like colony and downregulation of Calcofluor white binding, both indicating that cellulose biosynthesis was dramatically downregulated. On the other hand, the enhanced reddish colony morphotype on the Congo red agar plate and enhanced fluorescence on the Calcofluor white plate of strain *Z. mobilis* ZM401 expressing the ZMO1055_ZM4_ catalytic mutants in the EAL domain, E356A and E536A, suggested an even higher accumulation of cellulose triggered by the DGC activity of ZMO1055 (Figure 2B,C; Li, Xia, et al. 2023). Of note, upon overexpression of ZMO1055_ZM4_, a brown Congo red binding phenotype appeared temporarily within the first 24 h (Figure S2a). The molecular basis of this characteristic dye binding is currently unknown, but might be coupled to the DGC activity of ZMO1055_ZM4_ (Li, Xia, et al. 2023). These observations thus indicate a temporal switch between DGC and PDE catalytic activity as can be observed for other GGDEF‐EAL proteins (Kader et al. 2006; Kulesekara et al. 2006).

As the GGDEF domain can also function as a sensory domain to affect the catalytic activity of the downstream EAL domain (Christen et al. 2005), we subsequently also assessed whether the PDE activity of ZMO1055_ZM4_ is affected by mutations in the GGDEF domain. We did not investigate the GGAQF catalytic mutant as we previously showed that it does not possess a phenotype in our assay (Li, Xia, et al. 2023). To this end, we chose to substitute arginine R221 of the RxxD (I‐site) motif, part of the cyclic di‐GMP binding I‐site (Christen et al. 2006), and R228 of the RxGGDEF conserved motif by alanine. Mutations R221A and R228A introduced into ZMO1055_ZM4_ still caused a de‐flocculation phenotype; however, few flocs were retained. These data indicated that these mutations in the GGDEF domain affect the catalytic activity of the GGDEF or EAL domain of ZMO1055_ZM4_, but only to a minor extent. The impairment of the allosteric inhibitory I‐site by the R221A substitution might enhance the DGC activity of the GGDEF domain of ZMO1055_ZM4_ (Christen et al. 2006; Li, Xia, et al. 2023).

To evaluate whether the observed changes in the self‐flocculation morphology are due to differences in protein expression level or enzymatic activity, relative expression levels of ZMO1055_ZM4_ and mutants were assessed by Western blot analysis detecting the C‐terminal 6xHis‐tag on the proteins. The results showed that production of the ZMO1055_ZM4_ variants with R228A and E356A substitutions was impaired, but there was no correlation between expression level and self‐flocculation (Figure 2E). Therefore, the difference in flocculation and colony morphology is due to the amino acid substitutions affecting the PDE activity, thus causing no alteration, or even an increase in cellulose biosynthesis and self‐flocculation compared to the vector control.

### 3.3 Heterologous Expression of ZMO1055_ZM4_ and Its Mutants Affect Phenotypes in the *Salmonella typhimurium* UMR1 Motility/Sessility Model

The interaction of the ZMO1055 scaffold with other proteins in *Z. mobilis* ZM4 might alter its catalytic activity and/or performance of the EAL domain. In order to assess the effect of the apparent catalytic activities of ZMO1055_ZM4_ and its variants in a heterologous host where intermolecular protein–protein interactions most likely are substantially different, we chose to assess the effect of ZMO1055_ZM4_ and its variants in an alternative unrelated model on cyclic di‐GMP regulated phenotypes, rdar biofilm morphotype and flagella‐based swimming motility, in *S. typhimurium* UMR1 (ATCC 14028 Nal^r^ rdar_28°C_). To this end, the strain with a deletion in the phosphodiesterase required mainly for motility regulation, *S. typhimurium* UMR1 Δ*yhjH*, has been shown to be a sensitive model to simultaneously assess PDE activity by downregulation of rdar biofilm morphotype expression and upregulation of flagella‐based swimming motility (El Mouali et al. 2017). As addition of a 6x‐His‐tag can alter the functionality or degree of regulation of a protein, we first tested the effect of 6x‐His on ZMO1055_ZM4_ and ZMO1055_ZM401_ performance. Indeed, addition of 6x‐His reduced the apparent phosphodiesterase activity of ZMO1055, but did not blur the effect of the A526V mutation (Figure S2b). Subsequently, we then assessed the effect of the catalytic mutants E356A and E536A on the EAL domain activity of ZMO1055_ZM4_. Those mutants did not alter the wild type phenotype with respect to rdar morphotype expression indicative of the loss of PDE activity of ZMO1055_ZM4_. On the other hand, swimming motility was even more repressed upon expression of these protein mutants potentially indicative of a residual apparent DGC functionality (Figure 2F,G; Li, Xia, et al. 2023). Thus, the *Z. mobilis* and *S. typhimurium‐* based assays, although showing grossly a similar regulation of cyclic di‐GMP responsive phenotypes, possess a different sensitivity to monitor the DGC activity of ZMO1055_ZM4_.

Upon expression of the ZMO1055_ZM4_ GGDEF domain mutants in strain *S. typhimurium* Δ*yhjH*, expression of wild‐type ZMO1055_ZM4_ and its R221A mutant abolished the rdar phenotype. In congruency, these two strains displayed enhanced swimming motility, indicating that the mutations in the GGDEF domain of ZMO1055_ZM4_ had no effect on the apparent phosphodiesterase activity. However, even though the R228A mutant in the GGDEF domain maintained rdar phenotype expression as the vector control strain, it displayed significantly enhanced swimming motility (Figure 2F,G). These observations indicate again a differential sensitivity of the two biological assays in the same strain for apparent catalytic activities. This observation is not surprising as cyclic di‐GMP turnover proteins have been shown to act locally (Giacalone et al. 2018; Römling et al. 2013) and can be dedicated to distinct physiological processes. Such is the phosphodiesterase YhjH dedicated to flagella‐based motility (Le Guyon et al. 2015). Alternatively, the R228A mutation has a complex effect on the apparent catalytic activity of ZMO1055_ZM4_ in the *S. typhimurium* model.

### 3.4 PAS‐GGDEF‐EAL Domain Structure Is Required for ZMO1055_ZM4_ Functionality

On the basis of conserved domain analysis by alignment of homologous proteins and protein structural modelling by Phyre2 (Kelley et al. 2015; Liu et al. 2020), we concluded that ZMO1055 contains an N‐terminal PAS (Per‐Arnt‐Sim) domain (Xing et al. 2023). PAS domains display a high sequence diversity explaining why the PAS domain of ZMO1055 has not been recognised by standard amino acid sequenced‐based two‐dimensional BLAST search (Altschul et al. 1990). A PAS domain does not only function as a signal receiver and transducer domain but also dimerises GGDEF/EAL domain‐containing proteins upon signal perception, enabling or enhancing the catalytic activity (Schirmer 2016).

**FIGURE 3:**
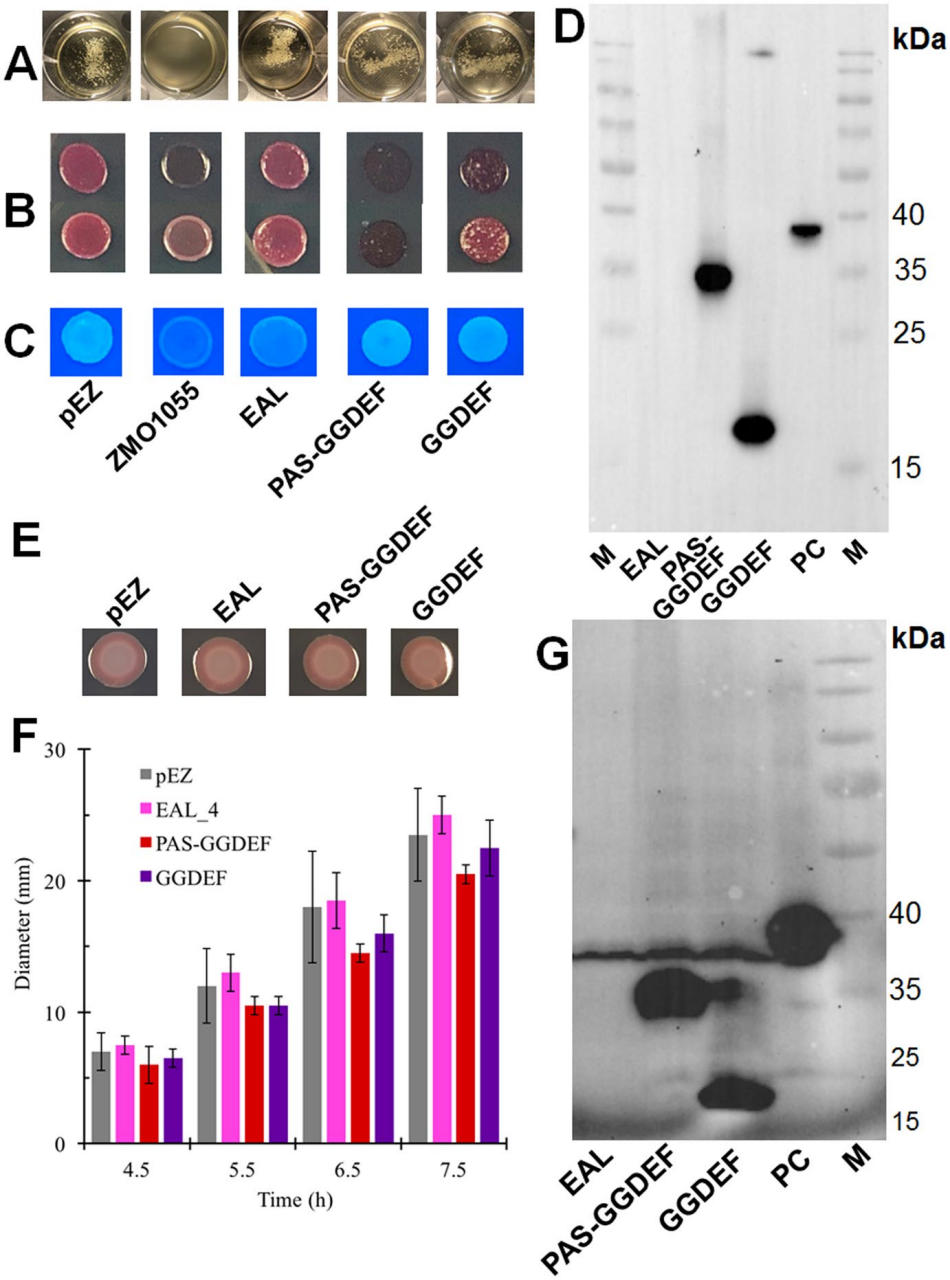
ZMO1055 of *Z. mobilis* ZM4 is a PAS‐GGDEF‐EAL domain protein with the full‐length domain structure required for activity. Flocculation (A), rdar morphotype (B) and Calcofluor white staining (C) assays of *Z. mobilis* ZM401 with overexpression of wild‐type protein ZMO1055_ZM4_ and variants truncated in individual domains. Production of the EAL, PAS‐GGDEF and GGDEF domain in *Z. motilis* ZM401 with the 6x‐His‐tagged patatin‐like phospholipase CapV as a positive control (D). Rdar colony morphology assay (E), motility (F) and protein production (G) of *S. typhimurium* Δ*yhjH* producing the EAL, PAS‐GGDEF and GGDEF domain of ZMO1055.

We assessed the impact of domain interactions for the activity of ZMO1055_ZM4_ by selective domain overexpression. As reported above, upon overexpression of full‐length ZMO1055_ZM4_ in the self‐flocculating strain ZM401, the self‐flocculation phenotype of ZM401 was completely disrupted, concomitant with substantial degradation of intracellular cyclic di‐GMP by ZMO1055_ZM4_ (Cao et al. 2022; Li, Xia, et al. 2023). We subsequently constructed different ZMO1055_ZM4_ variants with truncations of one or two domains. Overexpression of a ZMO1055_ZM4_‐derived construct containing only the EAL domain did not de‐flocculate *Z. mobilis* ZMO401, indicating the necessity of the PAS and/or GGDEF domain in assisting PDE activity, dimerisation and/or expression of ZMO1055_ZM4‐EAL_ (Figure 3A). Alternative phenotypic assays on Congo red and Calcofluor white plates indicated residual activity of ZMO1055_ZM4‐EAL_ compared with the vector control (Figure 3B,C). Western blot analysis demonstrated that expression of the stand‐alone ZMO1055_ZM4‐EAL_ was not detectable, indicating the requirement of the PAS and/or GGDEF domain or of additional amino acids immediate adjacent to the EAL domain for the stable expression of the EAL domain (Figure 3D).

We further investigated whether the GGDEF domain has a function on its own. Overexpression of either PAS‐GGDEF or GGDEF did not change the self‐flocculation phenotype compared with the vector control, suggesting that ZMO1055_ZM4‐GGDEF_ and ZMO1055_ZM4‐PAS‐GGDEF_ require the EAL domain for functionality in the self‐flocculation assay. However, the Congo red assay exhibited a substantial alteration in colony morphology and inhibition of cell growth by overexpression of the PAS‐GGDEF and GGDEF domain. Western blot analysis showed high expression of both constructs suggesting residual catalytic activity, GTP substrate binding of the GGDEF domain or other mechanisms to be responsible for this effect on cell physiology (Figure 3D; Li, Xia, et al. 2023).

Assessment of the functionality and expression of the individual domains in *S. typhimurium* Δ*yhjH* as an alternative and heterologous host showed no difference in rdar or motility morphology from vector control upon overexpression of the three constructs (Figure 3E,F). Thus, the truncated constructs are nearly inactive despite high expression of the PAS‐GGDEF and GGDEF domain also in the *Salmonella* system (Figure 3G). Again, ZMO1055_ZM4‐EAL_ was not expressed consistent with the production experiments in *Z. mobilis* ZM401.

### 3.5 Amino Acids at Position 526 Differentially Affect the Apparent Catalytic Activity of ZMO1055

The amino acid sequence similarity among GGDEF/EAL domains encoded by one genome is usually less than 35% with *Z. mobilis* ZM4 no exception from other bacterial species. Such shows the EAL domain of ZMO1055 33.1% and 25.6% identity to the EAL domain of ZMO401 and ZMO1487, respectively. Thus, we considered the possibility that A526, although not conserved among *Z. mobilis* ZM4 EAL domains (Figure 1B), possesses a specific role in one or more subgroups of EAL domains. We therefore analysed amino acid conservation at the equivalent of the position 526 of the EAL domain in ZMO1055_ZM4_ in the first 5039 non‐redundant most closely related proteins as derived from the NCBI and UniProt database (Altschul et al. 1990; Apweiler et al. 2004). After automatic and subsequently manual curation of the alignment of the EAL domains of these proteins, a statistical analysis of the conservation at this site was performed. Surprisingly, this analysis showed that among the 5039 sequences A526 is highly conserved in this ZMO1055 based subclass of GGDEF‐EAL domain proteins present in over 70.5% of the sequences and glycine present in 24.4%, while valine at position 526 is present at a frequency of 0.14% (Figure S3a). Other amino acids present were, for example, cysteine, with a frequency of 0.8%. The phylogenetic relatedness of the EAL domain of ZMO1055 was assessed by alignment with representative EAL domains. The phylogenetic tree indicates that the EAL domain of ZMO1055_ZM4_ forms a subclass distinct from other EAL domains (Figures 1D and S3b).

Above analyses and previous studies had shown that the A526V mutation in ZMO1055_ZM401_ maintains cell aggregation (flocculation) and the intracellular cyclic di‐GMP concentrations to a greater extent than ZMO1055_ZM4_ (Cao et al. 2022; Li, Xia, et al. 2023). The conservation of alanine at position 526 in subclass ZMO1055 GGDEF‐EAL domain proteins indicates that alanine is important for the functionality of the EAL domain. Thus, we raised the question of whether and to which extent replacement of alanine A526 by alternative amino acids affects the apparent catalytic activity of the EAL domain. The impact of amino acid substitutions was again assessed by the effect of the expression of ZMO1055 and its variants on the cyclic di‐GMP dependent phenotypes.

Alanine is the amino acid with the shortest non‐polar side chain of only one —CH_3_ group. The side chain of valine contains two additional non‐polar —CH_3_ groups. On the basis of the structural model of ZMO1055 and molecular docking simulations we have hypothesised that the increased size of the aliphatic side chain in valine allosterically affects the amino acid constellations in the catalytic centre and cyclic di‐GMP binding (Cao et al. 2022).

To extend this observation and to gain further insights into the molecular mechanism of the decreased apparent phosphodiesterase activity of ZMO1055_ZM401_, we consequently introduced alternative amino acid substitutions by site directed mutagenesis at the position 526 in order to investigate their effect on the apparent PDE activity of ZMO1055_ZM4_. Our choice of amino acids was based (1) on the frequency of occurrence of alternative amino acids in the first 5039 homologues of ZMO1055 (Figure S3a); and (2) on amino acids with similar chemical properties, but larger size of the side chain compared with alanine and valine. To this end, alanine was substituted by glycine, with 24.5% the second most frequent amino acid at position 526, which confers alpha helix destabilising properties. Furthermore, A526 had also been replaced by serine (frequency 2.4% at the 526 position), threonine (0.54%), isoleucine (0.04%) and leucine (not present but side chain with different branching compared to isoleucine). Serine and threonine are amino acids with a linear polar side chain, but larger size of the side chain when compared with alanine. Isoleucine and leucine possess a larger aliphatic side chain than alanine and valine.

**FIGURE 4:**
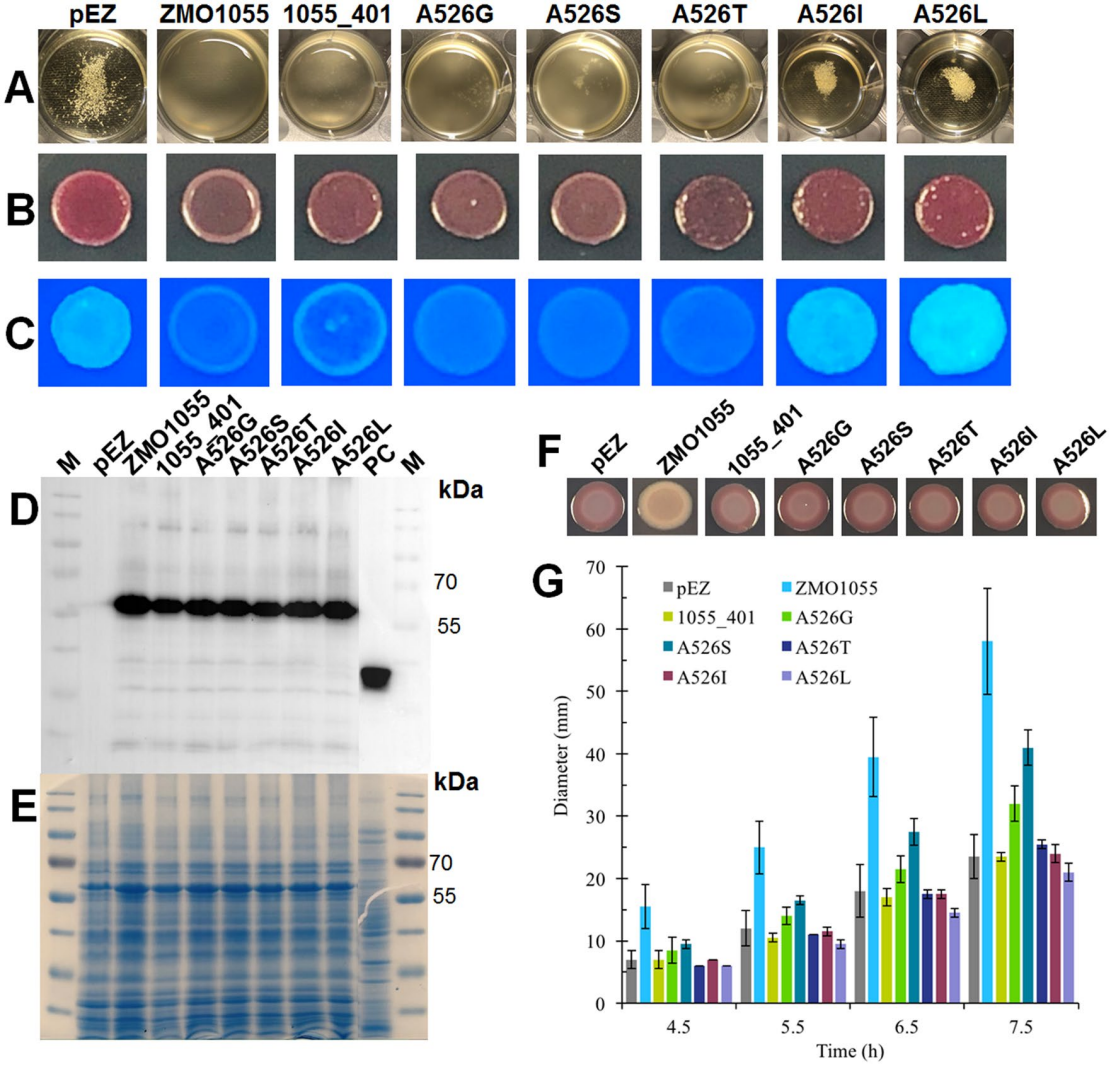
Size of the amino acid side chain at the A526 site of ZMO1055 regulates the apparent PDE activity. Flocculation (A) and rdar morphotype (at 48 h) (B) and Calcofluor white (at 24 h) (C) assays of *Z. mobilis* ZM401 overexpressing ZMO1055_ZM4_ or variants with the amino acid A526 substituted by valine, glycine, serine, threonine, isoleucine and leucine. Protein expression in *Z. mobilis* ZM401 was detected by Western blot (D) and SDS‐PAGE (E). Rdar phenotype (F) and swimming motility (G) of *S. typhimurium* UMR1 Δ*yhjH* strains expressing ZMO1055_ZM4_ or related protein variants.

We then assessed the effect of the amino acid substitutions with ZMO1055 variant expression in the *Z. mobilis* ZM401 and *S. typhimurium* UMR1 Δ*yhjH* model systems with the proteins ZMO1055_ZM4_ and ZMO1055_ZM401_ as references (Figure 4). In *Z. mobilis* ZM401 expressing ZMO1055_ZM4_ and its variants, substitution of alanine by glycine, serine, and threonine negatively affected the de‐flocculation activity compared to ZMO1055_ZM4_, to a similar extent as the substitution by valine, with few flocs remaining indicating diminished apparent PDE activity (Figure 4A). Of note, those amino acids either possess no side chain destabilising an alpha‐helix or a side chain in size up to valine. In steep contrast, overexpression of the protein variants with a leucine and isoleucine substitution not only retained but intensified self‐flocculation with tighter flocks and a clear supernatant exceeding flocculation of the vector control strain indicating highly reduced apparent PDE enzymatic activity and/or elevated DGC activity. Isoleucine and leucine possess a larger aliphatic side chain. As the self‐flocculation phenotype mediated by cellulose biosynthesis is associated with elevated concentration of intracellular c‐di‐GMP which is effectively degraded by ZMO1055_ZM4_ (Li et al. 2022; Li, Xia, et al. 2023), the size of the aliphatic side chain of the 526th amino acid significantly affects the apparent PDE activity most likely by steric hindrance with access of cyclic di‐GMP to the active site impaired (Cao et al. 2022).

Besides assessment of the effect of the amino acid substitutions at position 526 on the self‐flocculation phenotype, we assessed the effect of alterations in the apparent PDE activity of ZMO1055 by monitoring cellulose production by the Congo red and Calcofluor white binding assay in *Z. mobilis* ZM401. Upon overexpression, the reference proteins ZMO1055_ZM4_ and ZMO1055_ZM4_ A526V (ZMO1055_ZM401_) showed a characteristic Calcofluor white binding patterns with dye binding at the rim of the colony only. This observation indicates that the phosphodiesterase activity of ZMO1055 is highly active in late phase cells, but inactive in exponentially growing cells consistent with the temporal effect of ZMO1055 expression (Figure S2a). In contrast, overexpression of ZMO1055_ZM4_ containing either the A526S, the A526T and the A526G substitution showed equal low level Calcofluor white binding throughout the colony indicating still effective apparent PDE activity. We concluded that the mutations caused growth phase independent apparent phosphodiesterase activity of ZMO1055. The Congo red binding pattern of the agar grown colony upon overexpression of all three ZMO1055_ZM4_ variants followed this trend. These observations confirmed the hypothesis that the amino acid at position 526 affects the apparent phosphodiesterase activity through steric hindrance rather than by the chemical property of the amino acid side chain.

In contrast, overexpression of ZMO1055_ZM4_ with the A526I or A526L substitutions caused a reversion of the effect of the protein with dark red rough‐surfaced morphotypes and bright Calcofluor white binding higher than in the vector control strain (Figure 4B,C). In conclusion, the Congo red and Calcofluor white assay grossly confirmed the direction of the phenotypic consequences upon expression of the ZMO1055_ZM4_ mutants. In particular, the longer branched aliphatic side chains of the amino acids leucine and isoleucine substituting alanine at position 526 not only retained cellulose expression of *Z. mobilis* ZM401 but even enhanced the cellulose‐based phenotypes cell aggregation and dye binding capacity. These findings confirmed our hypothesis that amino acids at position 526 can affect the apparent PDE activity through longer aliphatic side chains causing steric hindrance rather than by chemical property. However, the minor effect of the A526G mutation still needs to be explained.

Western blot analysis was applied to assess the expression of ZMO1055_ZM4_ in comparison to its 526 position variants. When the total protein content based on assessment of the protein pattern after CBB G‐250 staining for a 12% SDS‐PAGE gel had been normalised and expression of ZMO1055_ZM4_ assessed, we concluded that production of variant proteins was not affected by the different mutations. Thus, the different self‐flocculation phenotypes were due to the altered apparent PDE activity of ZMO1055_ZM4_ variants rather than altered expression levels (Figure 4C–E).

Heterologous expression of the mutant proteins in the model of the rdar phenotype of *S. typhimurium* Δ*yhjH* was indiscriminatory as a nearly similar colony phenotype without any difference in colour or roughness as the vector control was observed (Figure 4F). On the other hand, swimming motility of *S. typhimurium* Δ*yhjH* strains showed a differentiated behaviour of the mutants: elevated motility was observed upon expression of ZMO1055_ZM4_ with the A526S and A536G substitution. However, the increase in motility did by far not reach the ZMO1055_ZM4_ level. Comparable to ZMO1055_ZM401_ (ZMO1055_ZM4_ A526V), expression of ZMO1055_ZM4_ A526T, A526I and A526L did not elevate swimming motility over the vector control in *S. typhimurium* Δ*yhjH* (Figure 4G). Therefore, the apparent PDE activity of ZMO1055_ZM4_ in *S. typhimurium* is affected by the 526th amino acid grossly depending on the size of the side chain.

### 3.6 Structural Model of the Effect of Amino Acids Predicted to Be Involved in Steric Hindrance in ZMO1055_ZM4_

**FIGURE 5:**
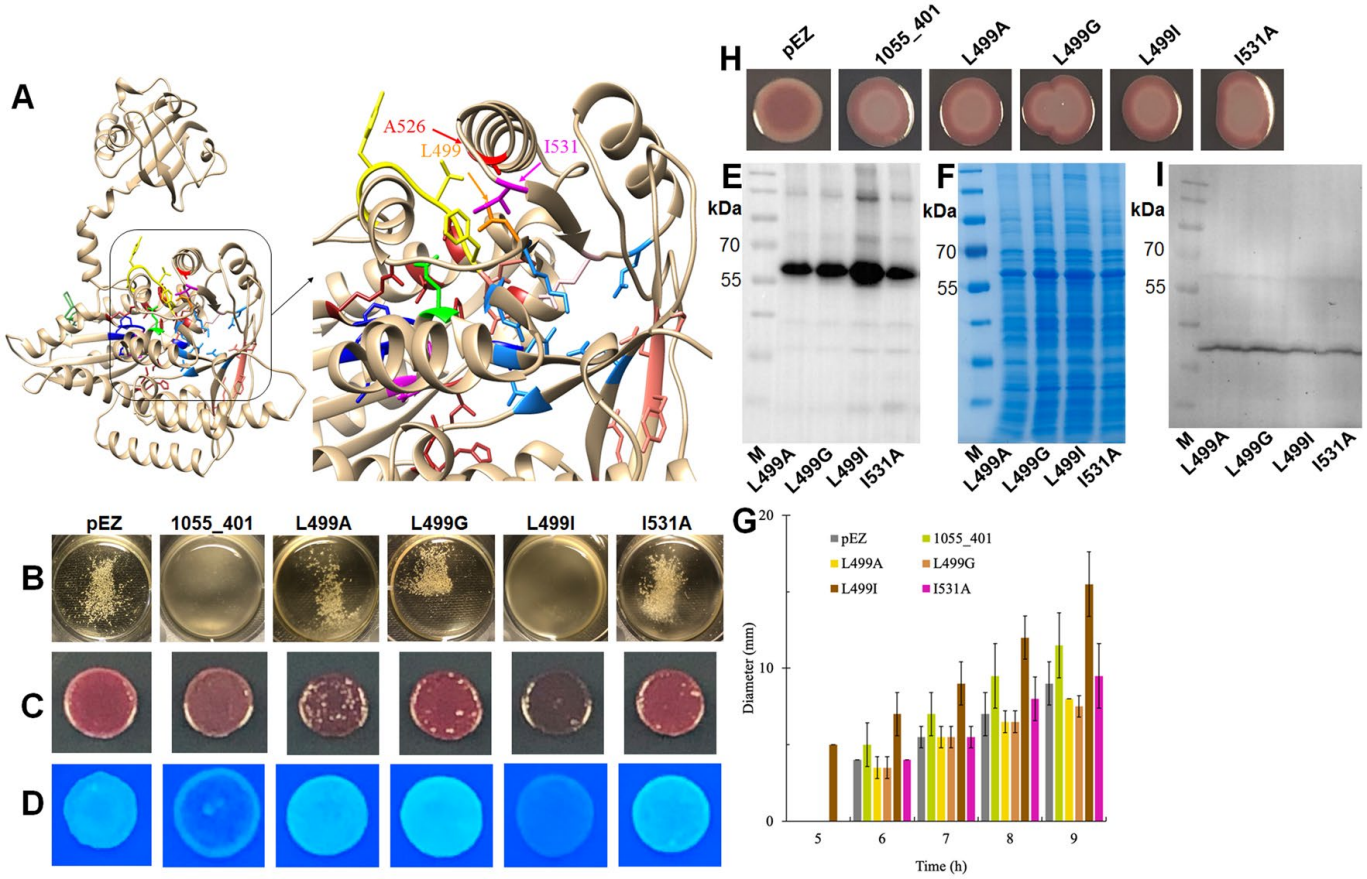
Possible mechanism applied by the mutation site A526 to affect apparent catalytic activity of ZMO1055. Structural modelling of ZMO1055_ZM4_ by Phyre2 (A). The mutation site (526th amino acid) in the EAL domain is coloured in red. In the catalytic site of the EAL domain indicates salmon the substrate binding amino acids, pink indicates the catalytic base and dodger blue indicates the Mg^2+^ binding amino acids. Yellow indicates Loop 6 and green Loop 6 stabilising amino acids. Orange indicates L499 and magenta indicates I531 mutated in the EAL domain. In the GGDEF domain, the substrate binding amino acids (firebrick), the Mg^2^^+^ binding amino acids (magenta), the allosteric I‐site amino acids (forest green) and the transition state stabilizing amino acids (dark blue) are also indicated. Flocculation (B), rdar (at 48 h) (C) and Calcofluor white staining (at 24 h) (D) assay of *Z*. *mobilis* ZM401 mutants with overexpression of ZMO1055_ZM401_ and its L499A, L499G and I531A variants. Protein expression of ZMO1055_ZM401_ by Western blot (E) and SDS‐PAGE (F). Swimming motility (G), rdar biofilm colony morphotype (H) and protein expression (I) of *S. typhimurium* Δ*yhjH* strains expressing ZMO1055_ZM401_ or respective protein variants.

A structural model of ZMO1055_ZM4_ was constructed by de novo calculation with Phyre2 and I‐TASSER as well as with SWISS‐MODEL with PA0861 (PDB: 5XGB.1.A) from *P. aeruginosa* PAO1 as the reference structure (Figure 5A and data not shown; Kelley et al. 2015). Similar structural characteristics such as the arrangement of secondary structures and locations of conserved sites were exhibited when comparing the three 3D structures of ZMO1055_ZM4_. The amino acid alanine 526 locates close to the end of an α‐helix with the aliphatic side chain facing towards the active centre of the EAL domain. However, the side chain of alanine 526 does not establish direct contact with active site residues, but is separated by two β‐strands formed by amino acids D497‐I501 and I531‐E536. We decided to test whether the apparently lower PDE catalytic activity of ZMO1055_ZM401_ (ZMO1055_ZM4_ A526V) could be recovered via relaxed side chain interactions involving amino acid residues of those two β‐sheets. On the basis of the structural models, the amino acids leucine L499 and isoleucine I531 were chosen to be substituted by amino acids with shorter side chains as they are in the line from A/V526 to the active centre (Figure 5A). Leucine and isoleucine constitute 96.3% of the amino acids present at position 499 and 45.1% at position 531 among the first 5039 homologues of ZMO1055.

We subsequently replaced L499 or I531 by alanine in ZMO1055_ZM401_, an amino acid with a smaller side chain. Only 0.1% of amino acids at position 531 are alanine. In addition, we replaced L499 by glycine and its isomer isoleucine, which has branching at the γ‐carbon instead of the δ‐carbon. The L499I mutation of ZMO1055_ZM401_ caused de‐flocculation of *Z. mobilis* ZM401 cell aggregates similar as ZMO1055_ZM401_ (Figure 5B), while the Congo red and Calcoflour white assay indicated higher apparent PDE activity. Contrary to predictions, overexpression of the ZMO1055_ZM401_ L499A and L499G variants, as well as the I531A variant in *Z. mobilis* ZM401, displayed flocculation exceeding the *Z. mobilis* ZM401 vector control, as well as enhanced Calcofluor white binding, indicating elevated cellulose production. Congo red binding was also enhanced, with distinct colouration upon overexpression of the variants (Figure 5C,D). Therefore, amino acids at positions 499 and 531 are involved in the regulation of the functionality of ZMO1055_ZM401_, with alanine at this position highly downregulating the apparent activity of the EAL domain, thereby showing a residual apparent diguanylate cyclase activity. Effects of amino acid substitutions at positions 499 and 531 on protein functionality are currently unpredictable by the structural model(s) and/or require refined structural models. We can, however, hypothesise that a distinct tight interaction between the aliphatic site chains of those proteins is required for catalytic activity, but lost upon the introduction of amino acids with other aliphatic side chains at the different positions in the protein.

Assessment of protein expression by Western blot analysis indicated that all ZMO1055_ZM401_ mutants had production levels equal to the parent protein ZMO1055_ZM401_ (Figures 5E,F, 4D,E).

When ZMO1055_ZM401_ and variants were heterologously expressed in *S. typhimurium* Δ*yhjH*, introduction of the ZMO1055_ZM401_ L499I mutant contributed to the stimulation of swimming motility to a higher extent than ZMO1055_ZM401_, while expression of the other double mutant constructs had no effect or even repressed swimming motility (Figure 5G). These results reflect the effect of the ZMO1055 EAL mutants in *Z. mobilis* ZM401. However, the rdar biofilm assay of *S. typhimurium* UMR1 Δ*yhjH* proved to be insensitive as it did not show any difference upon expression of the four proteins compared to ZMO1055_ZM401_ (Figure 5H). Production of proteins was similar in *S. typhimurium* UMR1 Δ*yhjH* (Figure 5I).

### 3.7 Effect of the A526V Mutation Is Also Displayed in Other GGDEF‐EAL Domain Proteins

**FIGURE 6:**
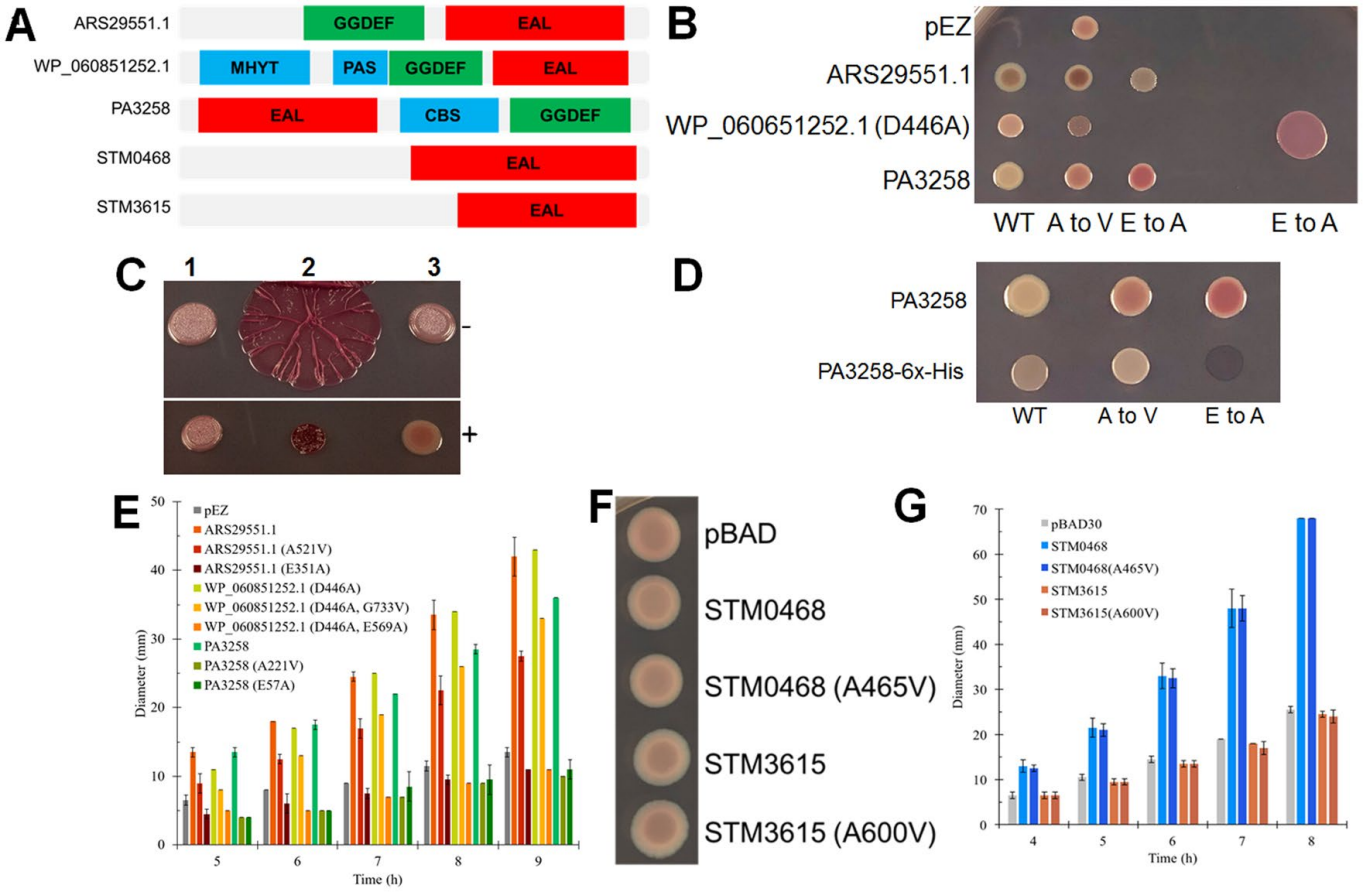
Effects of substitution of the equivalent of the A526 site on apparent PDE catalytic activity in GGDEF‐EAL or EAL proteins closely and more distantly related to ZMO1055. Domain structure of the five investigated EAL proteins (A); rdar colony morphology type (B) and flagella‐based swimming motility (E) of *S. typhimurium* UMR1 Δ*yhjH* strains expressing the selected GGDEF‐EAL proteins and its variants with A526V (A to V) and EAL>AAL (E to A) substitutions. Rdar colony morphology type upon overexpression of the GGDEF motif mutant WP_060851252.1 (D446A) versus wild type WP_060851252.1 in *S. typhimurium* UMR1 Δ*yhjH* (C) upon induction by 100 ng/L anhydrotetracycline. *S. typhimurium* UMR1 Δ*yhjH*: 1, vector control pEZ; 2, WP_060851252.1 cloned in pEZ; 3, WP_060851252.1 D466A cloned in pEZ. Effect of the C‐terminal 6xHis‐tag on the alteration of the colony morphology type of PA3258 overexpressed in the host *S. typhimurium* UMR1 Δ*yhjH* (D). Rdar colony morphology type (F) and flagella‐based swimming motility (G) of *S. typhimurium* UMR1 ΔyhjH expressing a *S*. *typhimurium* derived EAL (STM0468) and GGDEF‐EAL (STM3615) protein and their A526V equivalent variants. (B, D, F) Rdar colony morphology type has been monitored after 24 h of growth at 28°C on Congo red agar plates. (C) Flagella‐based swimming motility has been monitored at indicated time points with motility LB 0.25% agar plates.

To assess whether the effect of the A526V mutation on apparent PDE activity extends beyond ZMO1055 to be observed in other EAL‐domain containing proteins, we choose to investigate two more closely related GGDEF‐EAL proteins and three more distantly related EAL proteins which displayed an alanine at the position equivalent 526 of ZMO1055_ZM4_. After domain alignment and phylogenetic analyses, the two proteins chosen were ARS29551.1 and WP_060851252.1, GGDEF‐EAL proteins from *Sphingomonas* sp. KC8 and *Methylobacterium aquaticum* MA‐22A, respectively (Figure 6A). The EAL domains of these two proteins were 52.4% and 41.6% identical to the EAL domain of ZMO1055. A first protein with lower amino acid similarity but with an alanine at the position equivalent 526 is PA3258, an EAL‐CBS‐GGDEF protein of *P. aeruginosa* SG17M. Its EAL domain is only 24.3% identical to the EAL domain of ZMO1055. No phenotype had been detected for PA3258 in *P. aeruginosa* strains PAO1 and PA14 by transposon mutagenesis and gene over‐expression (Kulesekara et al. 2006), while in *Pseudomonas fluorescens* Pf0‐1 the homologous phosphodiesterase RapA depletes cyclic di‐GMP upon phosphate starvation (Newell et al. 2011). Two additional distantly related proteins with an alanine at a corresponding position in the EAL domain were STM0468 and STM3615 from *S.: typhimurium* ATCC 14028 (for convenience the nomenclature of *S. typhimurium* LT2 is used, with the gene cloned from the clonal variant *S. typhimurium* UMR1 [ATCC 14028 Nal^r^ rdar_28°C_]). To this end, the A to V amino acid substitution was introduced at the position corresponding to 526 of ZMO1055_ZM4_ by site‐directed mutagenesis.

All the five gene products and their A to V substitution mutants were expressed in the *S. typhimurium* UMR1 Δ*yhjH* model strain to assess their effects on the apparent PDE catalytic activity (Figure 6B–E). As substantial apparent diguanylate cyclase activity was observed for WP_060851252.1 (Figure 6C), the DGC activity was abolished by mutating the GGDEF motif to GGAEF (WP_060851252.1 [D446]) to prevent interference with the assessment of the PDE activity. When the wild type ARS29551.1 protein and WP_060851252.1 (D446A) were expressed in the *S. typhimurium* UMR1 Δ*yhjH* rdar biofilm model, the colonies appeared whitish compared with the pEZ vector control, confirming apparent PDE activity. However, when the A to V mutants were expressed, the colour of the colony turned more ‘reddish’ indicating elevated expression of biofilm extracellular matrix components. This effect suggested reduced apparent PDE activity of ARS29551.1 and WP_060851252.1 (D446A) consistent with the predictions (Figure 6B). Negative controls that abolished the catalytic activity of the EAL domain were constructed by site‐directed mutagenesis, replacing the glutamate by alanine in the EAL motif of ARS29551.1 and WP_060851252.1 (D446A). The rdar morphotype of ARS29551.1 with catalytically inactive EAL domain corresponded to the phenotype of the A521V mutant while production of WP_060851252.1 with the catalytically inactive EAL domain displayed a stronger rdar morphotype than its A724V mutant (Figure 6B).

Expression of the distantly related protein PA3258 C‐terminally tagged with 6xHis in *S. typhimurium* Δ*yhjH* showed an extreme and incongruent morphotype (Figure 6D). We reasoned that the 6xHis‐tag interferes with the protein functionality and/or stability. Upon expression of PA3258 without a 6xHis‐tag, a reduced rdar morphotype phenotype consistent with PDE activity and DGC activity (upon abolishment of the phosphodiesterase activity) was displayed (Figure 6B). Consistent with the other two proteins, the A221V mutation upregulated the rdar morphotype (Figure 6B).

Furthermore, stimulation of motility was diminished for all A to V mutants compared to their PDE competent wild‐type counterparts, hinting at substantial but mostly incomplete inhibition of the apparent PDE activity in ARS29551.1 and WP_060851252.1 (D446A) and a complete inhibition for PA3258 (Figure 6E). Upon overexpression of the PDE inactivated mutants, motility of *S. typhimurium* UMR1 Δ*yhjH* was even downregulated compared to the vector control in all instances, indicating residual apparent DGC activity of the proteins (Figure 6E).

The effect of an A to V substitution was also investigated in two other distantly related proteins, the EAL protein STM0468 and the GGDEF‐EAL domain protein STM3615 from *S. typhimurium* (Simm et al. 2007). An alanine is observed in distantly related EAL domain proteins even without a GGDEF domain. Even though STM0468 showed PDE activity in the motility assay, its A465V mutation did not show visible downregulation of the apparent catalytic activity (Figure 6F,G). STM3615 and its A600V mutant had no effect on motility, demonstrating that STM3615 does not have a phenotype in the *S. typhimurium* UMR1 Δ*yhjH* background as previously reported for the *S. typhimurium* UMR1 wild type (Figure 6F,G; Anwar et al. 2014; Simm et al. 2007). Furthermore, no change in apparent activity was observed upon expression between the wild type and the mutant protein upon assessment of the rdar morphotype (Figure 6H). In conclusion, the effect of the A to V mutation on apparent PDE activity is conserved in GGDEF‐EAL proteins ARS29551.1, WP_060851252.1 which belong to the ZMO1055‐related clade and in distantly related PA3258, but not in STM0468 which both possess a distantly related EAL domain.

### 3.8 Effect of Amino Acid 525 on the Apparent Phosphodiesterase Activity of ZMO1055

Upon analysis of the conservation of alanine at the 526th amino acid position in the aligned protein sequences, we noticed that only a few mainly non‐polar amino acids are present at the position 525 (Figure S3a). Methionine 525, as in ZMO1055, is present at a frequency of 31.8% in the first 5039 homologous proteins, while the frequency of leucine is 64.14% (Figure S3a). Therefore, methionine was substituted by leucine, constructing a M525L mutant in ZMO1055_ZM4_ to explore its effect on cyclic di‐GMP relevant phenotypes.

**FIGURE 7:**
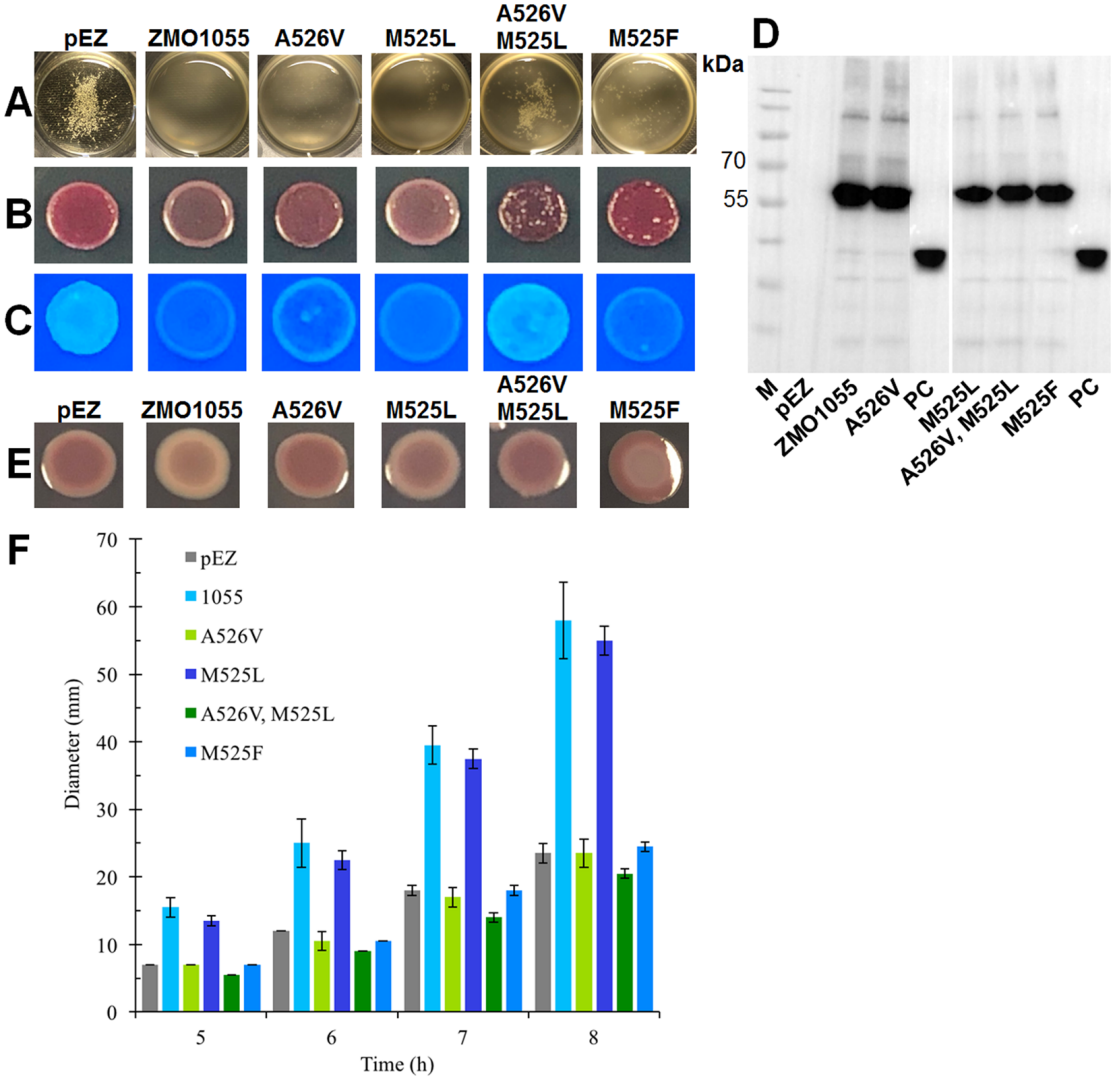
Effect of amino acid substitutions in the M_525_A motif of ZMO1055 on cyclic di‐GMP relevant phenotypes. Flocculation (A), rdar morphotype (at 48 h) (B) and Calcofluor white staining (at 24 h) (C) assays of *Z. mobilis* ZM401 with overexpression of ZMO1055_ZM4_ or its M_525_A motif substitutes. Western blot demonstrating expression of ZMO1055_ZM401_ mutants in *Z. mobilis* ZM401 (D). Rdar colony morphology biofilm (E) and swimming motility (F) of *S. typhimurium* UMR1 Δ*yhjH* strains expressing ZMO1055_ZM401_ or its M_525_A motif substitutes. (E) rdar colony morphology type has been monitored after 24 h of growth at 28°C on Congo red agar plates. (F) Flagella‐based swimming motility has been monitored at indicated time points with motility LB 0.25% agar plates.

Upon expression of ZMO1055_ZM4_ M525L in *Z. mobilis* ZM401, this protein variant diminished flocculation, rdar biofilm formation and Calcofluor white binding to a lesser extent than ZMO1055_ZM4_, but substantially more than ZMO1055_ZM401_, the ZMO1055_ZM4_ A526V mutant (Figure 7A–C). The M525L substitution in ZMO1055_ZM401_, however, retained substantial self‐flocculation and Congo red and Calcofluor white dye binding capacity compared to the ZMO1055_ZM401_ reference. Thus, the M525L substitution combined with A526V reduced the apparent PDE activity of ZMO1055 substantially (Figure 7). Leucine has a non‐polar branched side chain consisting only of methyl groups compared to methionine with a linear side chain. The *S. typhimurium* UMR1 Δ*yhjH* model was subsequently chosen to further assess the effect of the M525L substitution. The M525L mutant in ZMO1055_ZM4_ and ZMO1055_ZM401_ showed a similar trend with a diminished apparent PDE activity in *S. typhimurium* UMR1 Δ*yhjH* as upon expression in *Z. mobilis* ZM401 (Figure 7D,E). The M525L mutant of ZMO1055_ZM4_ and ZMO1055_ZM401_ even slightly reduced swimming motility compared to the parent proteins. In summary, as a hypothesis, the M525L amino acid substitution affects PDE activity predominantly through spatial arrangement of the side chain. AlphaFold 3 models of the wild type ZMO1055 and variant dimers, however, indicated that the side chain of the amino acid at position 525 is directed towards the interface of the antiparallel arranged alpha‐helices of the two monomers (Figure S4).

As the effect of the M525L substitution was observed, but not equally substantial in the ZMO1055 A526 as in the V526 sequence context, methionine at the 525th site was also substituted by phenylalanine, an amino acid present in 0.06% of the 5039 proteins most homologous to ZMO1055_ZM4_. Compared with ZMO1055_ZM4_ and ZMO1055_ZM4_ M525L, expression of ZMO1055_ZM4_ M525F in *Z. mobilis* ZM401 retained somewhat stronger self‐flocculation and highly reduced Calcofluor white staining, but surprisingly triggered a more pronounced rdar phenotype (Figure 7A–C). In conclusion, the amino acid at the 525th position affects the activity of ZMO1055 context dependent with respect to the relevant cyclic di‐GMP regulated phenotypes with the molecular mechanism still to be explained.

Expression of ZMO1055_ZM4_ M525F enhanced rdar morphotype expression compared to the vector control and had no effect on motility as expression of ZMO1055_ZM401_ (which is comparable to the vector control). These results showed that the ZMO1055_ZM4_ M525F mutant displayed an apparently highly impaired PDE activity in *S. typhimurium* Δ*yhjH* in contrast to *Z. mobilis* ZM401. In conclusion, substituting the amino acid not only at the 526th, but also at the 525th position affects the apparent PDE activity of ZMO1055. Exceptionally, the M525F substitution showed substantially divergent effects on phenotypes in the different (species) models.

## 4 Discussion

In this work, we investigated the impact of the A526V substitution in the PAS‐GGDEF‐EAL domain protein ZMO1055 which caused a significant downregulation of the apparent catalytic activity of its EAL domain phosphodiesterase associated with enhanced cellulose production and flocculation in *Z. mobilis* ZM4 (Cao et al. 2022; Li, Xia, et al. 2023). Our major findings include the observations that:

1. A526 is prevalent in the ZMO1055 subfamily of EAL domains, while valine at this position is infrequent.
2. Replacement of A526 by amino acids with a more bulky (aliphatic) side chain gradually causes downregulation of apparent phosphodiesterase activity most likely by allosteric hindrance of substrate access to the catalytic site.
3. The effect of the A526V and other amino acid substitutions in ZMO1055 is observed in different model systems.
4. The effect of the A526V and other substitutions is observed in subgroups of EAL domain proteins.
5. The increased size of the side chain of the amino acid at position 525 to possess an inhibitory effect on the apparent phosphodiesterase activity with the degree dependent on the sequence context and caused by a different mechanism.
6. The identity of the aliphatic side chain also at other amino acid positions in the EAL domain affects the apparent activity of ZMO1055.

Overall, this study shows the delicate regulation of cyclic di‐GMP signalling and its physiological output by only a single conserved amino acid substitution in the EAL domain outside of the signature amino acids in the PAS‐GGDEF‐EAL protein ZMO1055. Of note, the G165S amino acid substitution in the ZMO1055 ortholog of the evolved strain *Z. mobilis* F211 is determinative for cell flocculation and furfural tolerance (Hu et al. 2021). G165S is located in a non‐conserved amino acid sequence stretch in the GGDEF domain (Figure S1a).

Although non‐synonymous single nucleotide polymorphisms occur in open reading frames of bacterial isolates, clones and more frequently among bacterial clones, their impact on protein functionality and subsequently microbial physiology and metabolism has been rarely investigated, perhaps due to the scarcity of screenable output phenotypes. It has been observed though that single or multiple amino acid substitutions in cyclic di‐GMP turnover proteins outside of the conserved signature motifs can have a profound impact on readily screenable phenotypes like colony biofilms potentially through alteration of the catalytic activity of the domain, alterations in protein–protein interactions, alteration of signal perception and transduction or other mechanisms (Beyhan and Yildiz 2007; Cimdins‐Ahne et al. 2023; Cimdins et al. 2017; Gourinchas et al. 2017; Sokaribo et al. 2021). However, although single or multiple amino acid substitutions of cyclic di‐GMP turnover proteins are frequently observed in databases, effects of amino acid substitutions outside the conserved signature motifs are currently hardly predictable but require experimentation to assess their effect on relevant phenotypes.

In this context, amino acids with aliphatic side chains can be highly conserved in proteins to provide a functionality beyond a plain structural role. Although amino acids with aliphatic side chains have been shown to be involved in the high affinity binding of receptors to ligands such as cyclic di‐GMP (Roelofs et al. 2015; Wang et al. 2016) and the functionality of the CsgD biofilm transcriptional regulator (Wen et al. 2017), the impact of the choice of the different aliphatic side chains has been rarely explored (Baumann and Zerbe 2024). In this work, we demonstrate that amino acids with aliphatic side chains that differ by only one or two methyl groups and which are not directly located in the catalytic site can have a profound effect on the performance of the GGDEF‐EAL diguanylate cyclase/phosphodiesterase ZMO1055. We hypothesise, though, that a more determinative role of amino acids with aliphatic side chains extends beyond the position equivalent 526 in ZMO1055 to other positions and to other cyclic di‐GMP turnover proteins.

The phenotype to monitor the apparent catalytic activity of ZMO1055 has been predominantly self‐flocculation of *Z. mobilis* ZM4 caused by secretion of the exopolysaccharide cellulose. The exopolysaccharide cellulose, a 1,4‐beta‐D‐glucan, is a (biofilm) extracellular matrix component of microbes throughout the phylogenetic tree mediating cell‐substrate interactions such as adhesion under flow conditions and cell–cell interactions such as microcolony and biofilm formation and cell aggregation (flocculation) (Grantcharova et al. 2010; Jeon et al. 2012; Römling and Galperin 2015; Spiers et al. 2003; Williams and Cannon 1989; Xia et al. 2018). There exist at least five distinct cellulose biosynthesis gene clusters that can be discriminated by distinct accessory genes in the cluster (Römling and Galperin 2015). As a major regulatory mechanism, cyclic di‐GMP signalling post‐transcriptionally activates cellulose biosynthesis via the C‐terminal PilZ domain of the BcsA cellulose synthase (Morgan et al. 2014; Ryjenkov et al. 2006). However, cyclic di‐GMP signalling is not only tightly coupled to cellulose biosynthesis, but ubiquitously stimulates multicellular behaviour and alters the associated physiology and metabolism in bacteria (Albicoro et al. 2024; Chou and Galperin 2016; Mao et al. 2025). It is thus predicted that elevated cyclic di‐GMP levels caused by the diminished apparent phosphodiesterase activity of ZMO1055_ZM401_ and other aliphatic side chain variants can also affect other physiological traits besides cellulose biosynthesis.

Thus, not only flocculation based on cellulose production, but also the colony morphotype as a plate‐grown biofilm of *Z. mobilis* ZM401 and *S. typhimurium* UMR1 are sensitive biological models to assay the apparent catalytic activity and, potentially, the physiological and metabolic impact of cyclic di‐GMP turnover proteins (Simm et al. 2014). To our current knowledge, in *Z. mobilis* ZM401, self‐flocculation and the colony morphotype is based on solely cellulose biosynthesis, while in *S. typhimurium* UMR1, expression of the biofilm transcriptional regulator CsgD activates cellulose biosynthesis and amyloid fimbriae to commonly produce the cyclic di‐GMP dependent rdar morphotype with multiple targets for the second messenger. A diversity of colony morphotypes has been observed in this work for *Z. mobilis* ZMO401 though. This variability in morphotypes is reflected by the differential effect upon overexpression of ZMO1055 variants with the mostly conserved amino acids substitutions at the 525/526 site previously not implicated in functionality. On the other hand, flagella‐regulated swimming motility repressed by cyclic di GMP signalling is another sensitive biological assay to assess the activity of cyclic di‐GMP turnover proteins (Ryjenkov et al. 2006; Simm et al. 2004). In most instances, shifts in these biological assays were congruent for a ZMO1055 variant indicating that features intrinsic to the protein variants determine their functionality.

In the different bacterial species, cellulose biosynthesis is regulated by cyclic di‐GMP turnover proteins with diverse domain composition and cellular localisation which can be membrane‐bound or ‐associated or cytosolic thereby being localised or dispersed (Römling and Galperin 2015; Tal et al. 1998). Besides membrane‐bound enzymes such as the diguanylate cyclase AdrA, frequently, PAS‐GGDEF or PAS‐GGDEF‐EAL domain proteins provide the cyclic di‐GMP for the activation of cellulose biosynthesis (Liu et al. 2020; Römling et al. 2000; Tal et al. 1998). Thereby, the degree of activation of cellulose biosynthesis is not necessarily directly correlated with the cellular cyclic di‐GMP levels. The lack of correlation between the actual cyclic di‐GMP concentration and activation of cellulose biosynthesis by distinct enzymes shows the complex regulation of biochemical processes by second messenger signalling (Kader et al. 2006; Li, Xia, et al. 2023; Tal et al. 1998), although correlation between cyclic di‐GMP concentration and target output exists for a specific cyclic di‐GMP turnover protein (Massie et al. 2012). Local (close association of the diguanylate cyclase with the cellulose biosynthesis complex (Abidi et al. 2022; Kader et al. 2006; Massie et al. 2012)) and global signalling events might play a role. In the case of ZMO1055, the phosphodiesterase activity of the PAS‐GGDEF‐EAL domain protein is dominant in the *Z. mobilis* flocculation and *S. typhimurium* rdar morphotype and motility assay. However, residual DGC activity has been observed in different assays with single amino acid substitutions variants (Figures 2G and 4G) congruent with the demonstration that the ZMO1055's diguanylate cyclase activity promotes cellulose biosynthesis (Li, Xia, et al. 2023). Furthermore, the temporal appearance of a “brown” colony morphotype might indicate a temporal imbalance between the diguanylate cyclase versus the phosphodiesterase activity causing the activation of a novel matrix or cell membrane component alternatively indicate enhanced membrane permeability or other novel mechanistical cues that affect dye binding. Temporal alteration in the two opposite catalytic activities has been observed for other GGDEF‐EAL domain proteins throughout the growth phase such as STM3388 in *S. typhimurium* (Kader et al. 2006). Thus, abolishment of the diguanylate cyclase activity of ZMO1055_ZM4_ would have been an alternative experimental set‐up to assess the functionality of amino acid substitutions in the EAL domain.

Besides the direct functional impact of the A526V substitution, we observed that the ZMO1055 PAS‐GGDEF‐EAL protein in the phylogenetic context is subject to evolution. First of all, assessment of the 100 most homologous proteins indicated that the N‐terminal sensory PAS domain shows the highest amino acid sequence divergence (Figure S3b). It is, however, well known that sensory domains are usually subject to accelerated evolution.

The GGDEF domain in homologous proteins is also subject to evolution although to a lesser degree. Closest ZMO1055 homologues of members of the *Sphingomonas* genus are encoded at a conserved location on the chromosomes between *parC* encoding the DNA topoisomerase IV subunit A and *pcnB* coding for the poly(A)‐RNA polymerase I in members of the Zymomonadaceae and the closely related Sphingomonadaceae family in contrast to other alpha‐proteobacterial genera where the chromosomal context of ZMO1055 homologues is variable with homologues present as PAS‐GGDEF‐EAL or MHYT‐PAS‐GGDEF‐EAL proteins (Figure S3c,d). Like in ZMO1055, the GGDEF motif in several of these homologues is subject to change including the presence of GGDEL/M, AGDEF, GADEF, AADEF, GGNEF and variants, suggesting alterations in the degree of the catalytic activity, substrate specificity and/or loss of catalytic activity (Figure S3b). Of note, GGDEF domains with a substitution of the first glycine to serine in the GGDEF motif have been shown to still possess catalytic activity (Cimdins‐Ahne et al. 2023; Hunter et al. 2014; Perez‐Mendoza et al. 2011) equally as a domain with the mutant GGQEF motif (Li, Xia, et al. 2023). We therefore considered a GGDEF domain with a GGDEL/M and AGDEF motif to still possess (restricted) catalytic activity; AADEF and GGNEF motif domains were preliminary categorised as non‐active. In any case, an alteration in the GGDEF motif is not entirely predictive for a lack of catalytic activity.

While the EAL domain is predicted to be catalytically active in the vast majority of homologues (Figure S3d,e), the A526S, A526T, A526V, and A526I substitutions as present in ZMO1055 homologues can be considered as a first, still readily revertible, evolutionary step towards a gradually reduced activity of the EAL domain which eventually results in a catalytically incompetent domain. However, the impact of the A526 amino acid substitution might be specific to the ZMO1055 clade of EAL domains as it can require a specific backbone constellation. Of note, the sequence of distantly related EAL domain clades, such as the ZMO1487‐based clade, is substantially different (Figure S3e). Eventually, ZMO1055 homologues can develop into proteins with catalytically incompetent GGDEF and EAL domains.

## Author Contributions

**Feng‐wu Bai:** supervision, funding acquisition, writing – review and editing, resources. **Ute Römling:** conceptualization, investigation, funding acquisition, writing – original draft, methodology, validation, visualization, formal analysis, project administration, data curation, supervision, resources. **Lian‐Ying Cao:** conceptualization, investigation, formal analysis, writing – review and editing. **Xue Zhang:** investigation, writing – review and editing.

## Funding

This work was supported by Chinese Scholarship Council. Vetenskapsrådet, diary number 2022‐04865.

## Conflicts of Interest

The authors declare no conflicts of interest.

## Supporting information


**Data S1:** mbt270308‐sup‐0001‐supinfo.docx.

**Data S2:** mbt270308‐sup‐0002‐Tables S1‐S3.docx.

## Data Availability

The data that supports the findings of this study are available in the Supporting Information of this article.
