# Supplementary material for: Previously Uncharacterised Aliphatic Amino Acid Positions Modulate the Apparent Catalytic Activity of the EAL Domain of ZMO_1055 and Other Cyclic Di‐GMP‐Specific EAL Phosphodiesterases
Source: Microb Biotechnol. 2026 Feb 12;19(2):e70308. doi: 10.1111/1751-7915.70308 (PMC12900916; doi:10.1111/1751-7915.70308)
Supplement: Supplementary file 1 — Data S1: mbt270308‐sup‐0001‐supinfo.docx. [file MBT2-19-e70308-s001.docx]

**Supporting Information**

**Previously uncharacterized aliphatic amino acid positions modulate the apparent catalytic activity of the EAL domain of ZMO_1055 and other cyclic di-GMP specific EAL phosphodiesterases**

Lianying Cao^1,2^, Xue Zhang^2,#^, Feng-wu Bai^2^, Ute Römling^1*^

^1^Department of Microbiology, Tumor and Cell Biology, Biomedicum, Karolinska Institutet, 171 77 Stockholm, Sweden

^2^State Key Laboratory of Microbial Metabolism, Joint International Research Laboratory of Metabolic & Developmental Science, and School of Life Science and Biotechnology, Shanghai Jiao Tong University, Shanghai 200240, China

^#^, current address: Zhejiang Key Laboratory of Multiomics and Molecular Enzymology and Department of Biotechnology and Biomedicine, Yangtze Delta Region Institute of Tsinghua University, Zhejiang, 314006, China

*Corresponding author: [ute.romling@ki.se](mailto:ute.romling@ki.se); Phone: 0046-8-52487319

**Supplementary material**

**Title page 1**

**Index 2**

**Supplementary information 3**

**Experimental procedures 3**

**Supplementary Figure 1 5**

**Supplementary Figure 2 6**

**Supplementary Figure 3 7**

**Supplementary Figure 4 14**

**Supplementary Table S1: Strains used in this study**

**Supplementary Table S2: Plasmids used in this study**

**Supplementary Table S3: Primers used in this study**

**Supplementary information**

**Experimental procedures**

Protein sequences used for the alignment of GGDEF domain proteins in Figure 1a were: The sequences used in the alignment are PA0861_PSEAI (*Pseudomonas aeruginosa*); PA0575_PSEAI (*P. aeruginosa*); PleD_CAUCR, Q9A5I5 (*Caulobacter crescentus*); WspR_PSEAI, Q9HXT9 (*P. aeruginosa*); ARS29551.1 (A0A1X9YLB7; *Sphingomonas* sp. KC8); WP_084652956.1 (A0A1S1HAE2; *Sphingomonas haloaromaticamans*); TMJ20004.1 (A0A537MIH2; α-proteobacterium); ABQ66480.1 (A0A9J9H7P0; *Rhizorhabdus wittichii* DSM6014); TQL16391, (FBY51_1449, *Z. mobilis* NRRL B-4492); PROMIR_GGDEF (B3F0R5; *P. mirabilis*); STRGAL_GGDEF (WP_012961431.1; *S. gallolyticus* UCN34), YdeH_ECOLI, P31129 (*E. coli*); AdrA_SALTY, Q9L401 (*S. typhimurium*); PA4332_PSEAE, Q9HW69 (*P. aeruginosa*); DgcB_CAUCR, A0A0H3CAN8 (*C. crescentus*); CD1420_Cdif, Q18BU4 (*Clostridium difficile*); VCA0965_VIBCH, Q9KKY5 (*Vibrio cholerae*); ECA3270_PECAS, Q6D226 (*Pectobacterium atrosepticum*); GSU1658_GEOSL, Q74CL4 (*Geobacter sulfurreducens*); MXAN_2643, Q1D911 (*Myxococcus xanthus*); YciR_SALTY, A0A0F6B1Y8; DGC1_KOMXY, O87374 (*Komagataeibacter xylinus*); Y1354_MYCTU Rv1354c/P9WM13 (*Mycobacterium* *tuberculosis*); SE_0528_STAES, Q8CTF5 (*S. epidermidis*) and BifA_PSEAE, (Q9HW35; *P. aeruginosa*). In VCA0965, the sequence ‘RATNQHDY’ was deleted to optimize the alignment.

Protein sequences used for the alignment of EAL domain proteins in Figure 1b were: MorA_PSEAI (*P. aeruginosa*); PA0575_PSEAI (*P. aeruginosa*); RocR_PSEAI, Q9HX69 (*P. aeruginosa*); YciR_SALTY, A0A0F6B1Y8 (*S. typhimurium*); PDEA3_KOXYL, O87378 (*K. xylinus*); ARS29551.1 (A0A1X9YLB7; *Sphingomonas* sp. KC8); WP_084652956.1 (A0A1S1HAE2; *Sphingomonas haloaromaticamans*); TMJ20004.1 (A0A537MIH2; α-proteobacterium); ABQ66480.1 (A0A9J9H7P0; *Rhizorhabdus wittichii* DSM6014); TQL16391, (FBY51_1449, *Z. mobilis* NRRL B-4492); YahA_ECOLI, P21514 (*E. coli* K-12); YhjH_SALTY, A0A0F6B886 (*S. typhimurium*); YE2225_YENTE, A1JQ37 (*Yersinia enterocolitica*); DGC2_Komxyl, O87377 (*K. xylinus*); LapD_PSEFL, Q3KK31 (*P. fluorescens* Pf0-1); FimX_PSEAI, Q9HUK6 (PA4959; *P. aeruginosa*); STM1344_SALTY, D0ZW85 (*S. typhimurium* ATCC14028); ToxR_PSEAI, P09852 (*P. aeruginosa*); CsrD_ECOLI, P13518 (*E. coli* K-12).

Protein sequences used for the alignment of GGDEF and EAL domain proteins in Figure 1c and 1d were:

WP_128452934.1 (*Sphingomonas* sp. UV9); RMB53780.1 (*Sphingomonas* sp. PP-CE-3A-406); RKE50106.1 (*Sphingomonas* sp. PP-CC-1A-547); WP_042466648.1 (*Sphingomonas* sp.); WP_043060651.1 (*Sphingomonas melonis*); WP_146571662.1 (*Sphingomonas panacisoli*); WP_052192376.1 (*Sphingomonas* sp. 3F27F9); WP_010215794.1 (*Sphingomonas* sp. PAMC26621); WP_199857419.1 (*Sphingomonas* sp. EC-HK361); VVT12883.1 (*Sphingomonas* sp. EC-HK361); TCP36799.1 (*Sphingomonas* sp. BK235); WP_183919820.1 (*Sphingomonas* sp. BK069); WP_185209380.1 (*Sphingomonas* sp. NBWT7); OWK33834.1 (*Sphingomonas dokdonensis*); KKI18272.1 (*Sphingomonas* sp. *Ag1*); WP_223030959.1 (*Hephaestia mangrovi*); WP_119035448.1 (*Hephaestia caeni*); OQW45249.1 (*Proteobacteria bacterium SG_bin6*); WP_184055079.1 (*Sphingomonas aerophila*); MBB4085960.1 (*Sphingomonas carotinifaciens*); WP_133497202.1 (*Stakelama pacifica*); WP_184003171.1 (*Stakelama sediminis*); MBN2971714.1 (*Roseomonas aeriglobus*); WP_165326902.1 (*Sphingosinithalassobacter tenebrarum*); WP_184084453.1 (*Sphingomonas xinjiangensis*); WP_082596657.1 (*Sphingomonas* sp. *Root241*); KRC82426.1 (*Sphingomonas sp. Root241*); WP_184168250.1 (*Sphingomonas kyeonggiensis*); MBO9712769.1 (*Sphingomonas sp.*); WP_192088648.1 (*Sphingomonas sp.* CFBP 13720); WP_206429212.1 (*Sphingomonas* sp. 2R-10); WP_082450548.1 (*Sphingomonas* sp. Leaf22); WP_084689592.1 (*Sphingomonas changbaiensis*); WP_167398520.1 (*Blastomonas natatoria*); GGB63084.1 (*Blastomonas aquatica*); MBK8373390.1 (*Sphingomonadales bacterium*); MBB6123217.1 (*Sphingobium subterraneum*); WP_214625022.1 (*Sphingobium nicotianae*); MBB5970805.1 (*Sphingobium* sp. B12D2A); WP_184474142.1 (*Rhizorhapis suberifaciens*); WP_167303492.1 (*Sphingobium vermicomposti*); WP_097091911.1 (*Sphingomonadales*); WP_103001363.1 (*Sphingobium* sp. SA916); WP_176590902.1 (*Sphingobium* sp. EM0848); KEQ54209.1 (*Sphingobium chlorophenolicum*); WP_048939139.1 (*Sphingobium yanoikuyae*); QNG45688.1 (*Sphingobium yanoikuyae*); PZU67968.1 (*Sphingobium* sp.); MBA4090039.1 (*Sphingobium* sp.); WP_066601729.1 (*Sphingobium*); WP_037477082.1 (*unclassified Sphingobium*); WP_188064543.1 (*Sphingobium* sp. KCTC 72723); WP_066765922.1 (*Sphingobium* sp. CCH11-B1); WP_031297476.1 (*Sphingobium lactosutens*); PDH66261.1 (*Sphingomonadaceae bacterium* MED-G03); ODU20494.1 (*Sphingomonas* sp. SCN 67-18); WP_145152192.1 (*Sphingomonas solaris*); WP_166040430.1 (*Sphingosinicella* sp. YJ22); WP_147042051.1 (*Sphingosinicella ginsenosidimutans*); WP_106638825.1 (*Allosphingosinicella vermicomposti*); MBV9932410.1 (*Alphaproteobacteria bacterium*); WP_109270337.1 (*Allosphingosinicella humi*); WP_218633346.1 (*Sphingomicrobium* sp. B8); WP_118857534.1 (*Sphingomonas mesophila*); WP_187714814.1 (*Sphingomonas daechungensis*); MBE1481263.1 (*Sphingomonas* sp. OAS965); WP_167105499.1 (*Sphingomonas oligoaromativorans*); NIJ34173.1 (*Sphingomonas oligoaromativorans*); WP_110156973.1 (*Nostoc* sp. 3335mG*.*); ARS29551.1 (*Sphingomonas* sp. KC8); ARS29551.1 (*Sphingomonas* sp. KC8); WP_010124830.1 (*Sphingomonas* sp. KC8); WP_089219786.1 (*Edaphosphingomonas laterariae*); WP_015459733.1 (*Sphingomonas* sp. MM-1); AGH50765.1 (*Sphingomonas* sp. MM-1); WP_084652956.1 (*Edaphosphingomonas*); WP_167072910.1 (*Sphingomonas vulcanisoli*); WP_171744425.1 (*Sphingomonas* sp. AP4-R1); WP_169943874.1 (*Sphingomonas lacunae*); WP_033074760.1 (*Sphingopyxis* sp. MWB1); MBN8845402.1 (*Sphingomonadales bacterium*); MBW8294942.1 (*Sphingopyxis* sp.); MBJ7439191.1 (*Sphingopyxis* sp.); WP_203235672.1 (*Jacksonvillea* sp. ISTCYN1); API60907.1 (*Tardibacter chloracetimidivorans*); WP_218445300.1 (*Pacificimonas pallii*); WP_147423396.1 (*Sphingosinicella microcystinivorans*); WP_066959033.1 (*Sphingomonadales*); WP_011240894.1 (*Zymomonas mobilis*); ZMO_1055_WP_011240894.1 (*Zymomonas mobilis*); WP 060851252.1 (*Methylobacterium aquaticum* MA-22A); WP_154738706.1 (*Hyphomicrobium* sp. Xq); WP_011241261.1 (*Zymomonas mobilis*); WP_011240318.1 (*Zymomonas mobilis*); AAD19713.1 (*Zymomonas mobilis*); WP_011240778.1 (*Zymomonas mobilis*); WP_011241154.1 (*Zymomonas mobilis*).

Protein sequences used in the phylogenetic tree of Figure S3b were: MBP2137887.1, *Sphingomonas* *echinoides* BE319; WP 010215794.1, *Sphingomonas* sp. PAMC 26621; SDA14076.1, *Sphingomonas* sp. NFR15; MBN8814462.1; *Sphingomonas* sp. SCN18_26_2_15_R5_F_65_27; OQW73042.1, Proteobacteria bacterium ST_bin13; MBA4773209.1, *Sphingomonas* sp. MCMED-G21; TCP36799.1, *Sphingomonas* sp. BK235 EV292_101297; OWK33834.1, *Sphingomonas dokdonensis*; KKI18272.1, *Sphingomonas* sp. Ag1; PKP94256.1, HGW-Alphaproteobacteria-16; KRC82426.1; *Sphingomonas* sp. Root241; PVX62630.1, *Sphingomonas* sp. CF311; WP 197418082.1, *Sphingomonas* sp. CCH13-B11; PZO75509.1, *Sphingomonas hengshuiensis*; KQM18664.1, *Sphingomonas* sp.; WP 171744425.1, *Sphingomonas* sp. AP4-R1; WP 167072910.1, *Sphingomonas vulcanisoli*; TMJ20004 1, Alphaproteobacteria bacterium AP_24; MBE1481263.1; *Sphingomonas* sp. OAS965; ARS29551 1 SPH KC8, *Sphingomonas* sp. KC8; WP 084652956 1 SPHHAL; *Sphingomonas*; ABQ66480 1 SPHWIT; *Sphingomonas wittichii* RW1; WP_013933534.1 (F8EU54, *Zymomonas mobilis* subsp. pomaceae ATCC29192) and WP 163642225.1, *Methylobacterium* sp. BTF04; WP 056485266.1, *Methylobacterium* sp. Leaf117; TNC10695.1. *Methylobacterium* sp. 17Sr1-39; WP 060851252.1, *Methylobacterium aquaticum*; WP 090962595.1, *Aureimonas phyllosphaerae*; SPZ47856_PSEAI.1, *Agrobacterium tumefaciens* biovar 1; RYD45316.1, Sphingomonadales bacterium; MBO6856075.1, *Roseibium* sp. JJ875_04115; GBE42618.1, bacterium *BMS3Bbin10;* WP_154738706.1, *Hyphomicrobium* sp. Xq; MBL8709276.1, *Rhodospirillaceae* bacterium; WP 133292192.1, *Dankookia rubra* PRK10060 and STM3615, *Salmonella typhimurium* ATCC14028; STM0468, S. *typhimurium* ATCC14028; PA3258, *P. aeruginosa* PAO1.

**Figures**


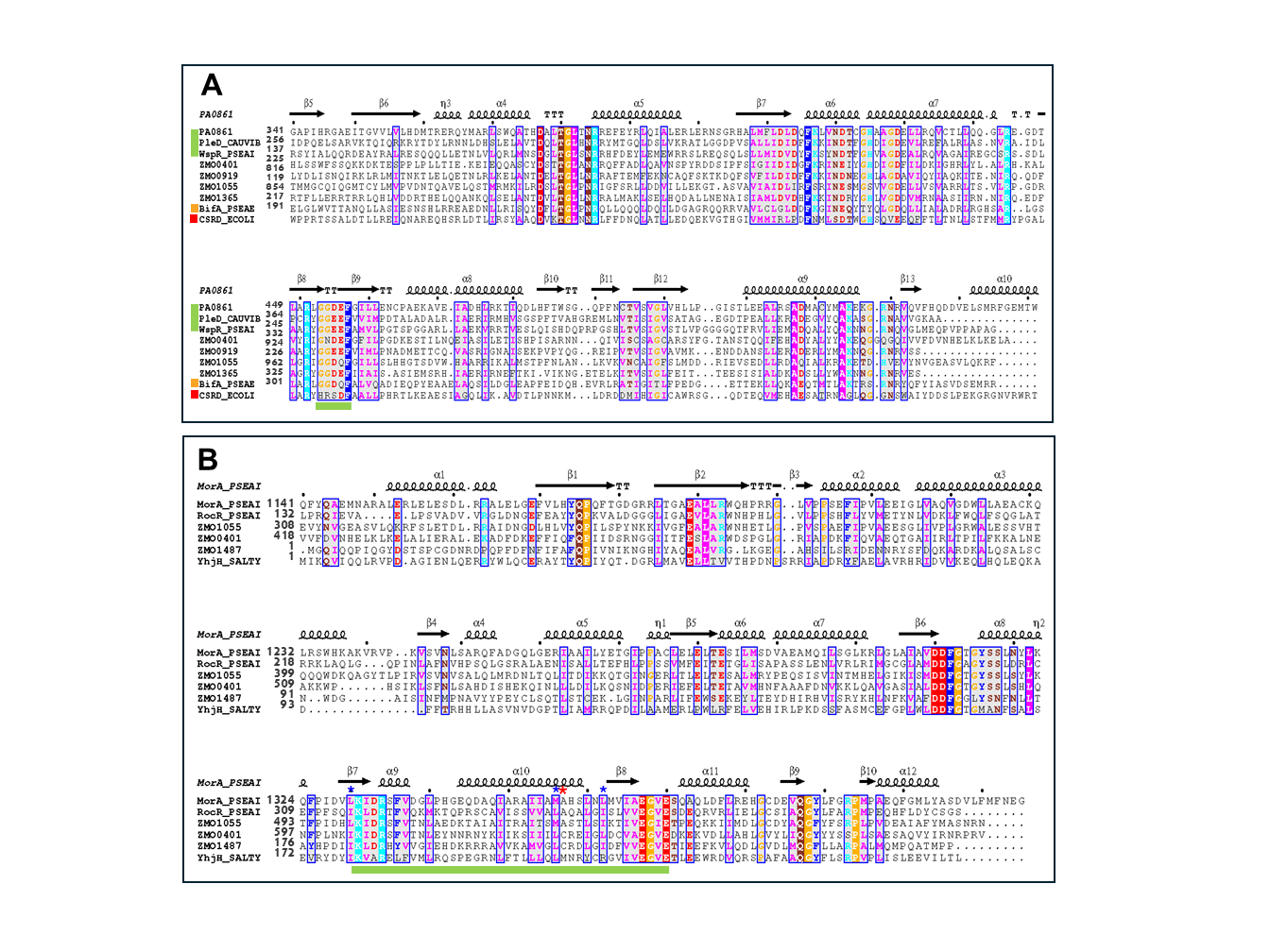


Figure S1: Alignment of the GGDEF and EAL domains encoded by the *Zymomonas mobilis* ZM4 genome with catalytically functional reference GGDEF and EAL domain proteins.

Figure S1a: Alignment of all GGDEF domains of *Z. mobilis* ZM4 with template and reference GGDEF domains. The GGDEF domain of the diguanylate cyclase PA0861 of *P. aeruginosa* (PDB: 5XGD) has been used for secondary structure designation. Green side bar indicates catalytically active domains (PleD and WspR), yellow side bar catalytically functional with C-terminal EAL domain (BifA) and red side bar catalytically inactive domains (CsrD). Underlined by a green bar is the GG(D/E)EF catalytic motif.

Figure S1b: Alignment of all EAL domains of *Zymomonas mobilis* with reference EAL domains. The EAL domain of the phosphodiesterase MorA of *P. aeruginosa* (PDB: 4RNI) has been used for secondary structure designation. RocR and the phosphodiesterase YhjH were added as comparison representing class I and class II phosphodiesterases (see Figure 1 a and b for explanation). All reference EAL domains are active phosphodiesterases. Underlined in green is the amino acid sequence between the conserved K(I/L/V)D and the EG(V/I)E motif that contains A526 (indicated by a green star). Blue stars indicate amino acids 499, 525 and 531 substituted in the course of this work.

Alignment by ClustalX 2.1 (Higgins and Sharp 1988) and visualization by ESPript 3.0 (Robert and Gouet 2014).


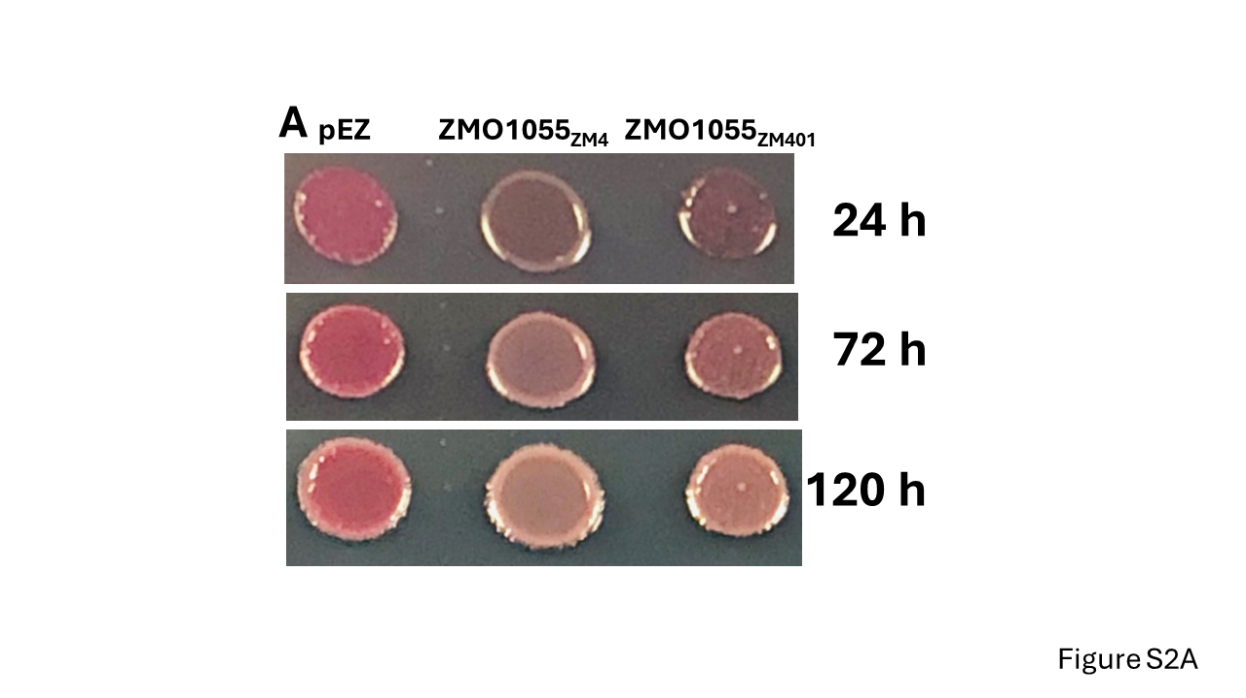


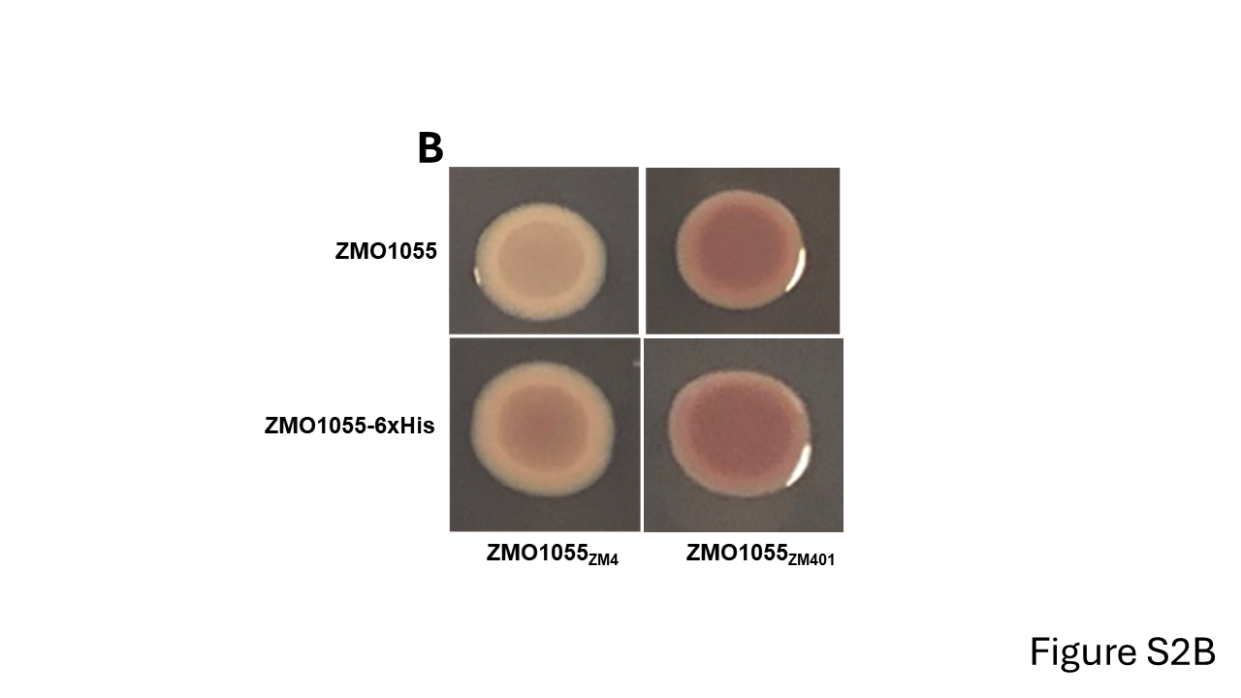


Figure S2: Colony morphologies of *Z. mobilis* ZM401 upon prolonged incubation up to 72 h (a) and effect of 6xHis C-terminal of ZMO1055 on colony morphology of *S. typhimurium* UMR1 Δ*yhjH* (b). (a) *Z. mobilis* ZM401 with pEZ vector control, ZMO1055_ZM4_ and ZMO1055_ZM401_ cloned in pEZ. Cells were grown on Congo red Rich-medium (RM) agar plates at 28°C for indicated time points. (b) *S. typhimurium* UMR1 Δ*yhjH* with ZMO1055_ZM4_ and ZMO1055_ZM401_ cloned in pEZ without and with a C-terminal 6xHis-tag. Cells were incubated on Congo red LB without salt agar plates at 28°C for 24 h.


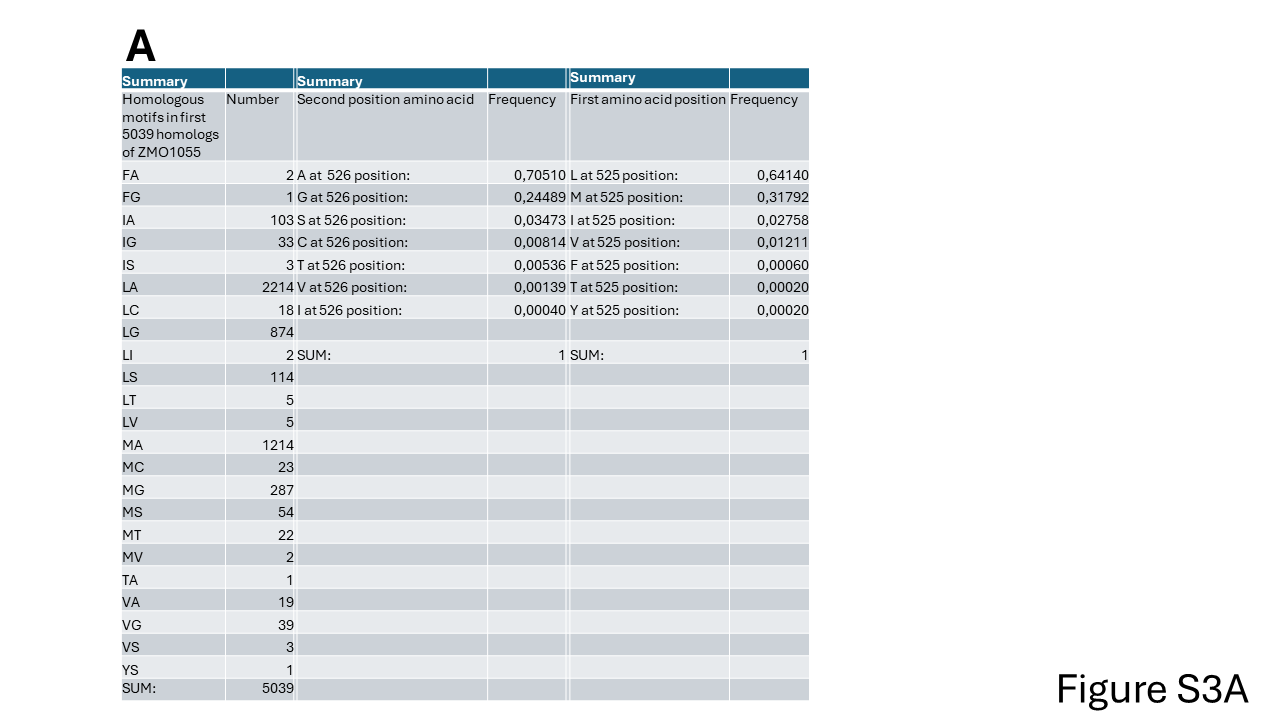


Figure S3: Bioinformatic analysis of the PAS-GGDEF-EAL protein ZMO1055_ZM4_ in the context of the most similar homologs.

Figure S3a: Frequency of amino acids at the M_525_A_526_ position. The 5039 most similar non-redundant ZMO1055_ZM4_ homologs were retrieved by Blast (April 2021) (Altschul et al. 1990), aligned with Clustal 2.1 (Higgins and Sharp 1988) with subsequent manual curation and the frequency of distinct amino acids at the M_525_A_526_ position calculated.


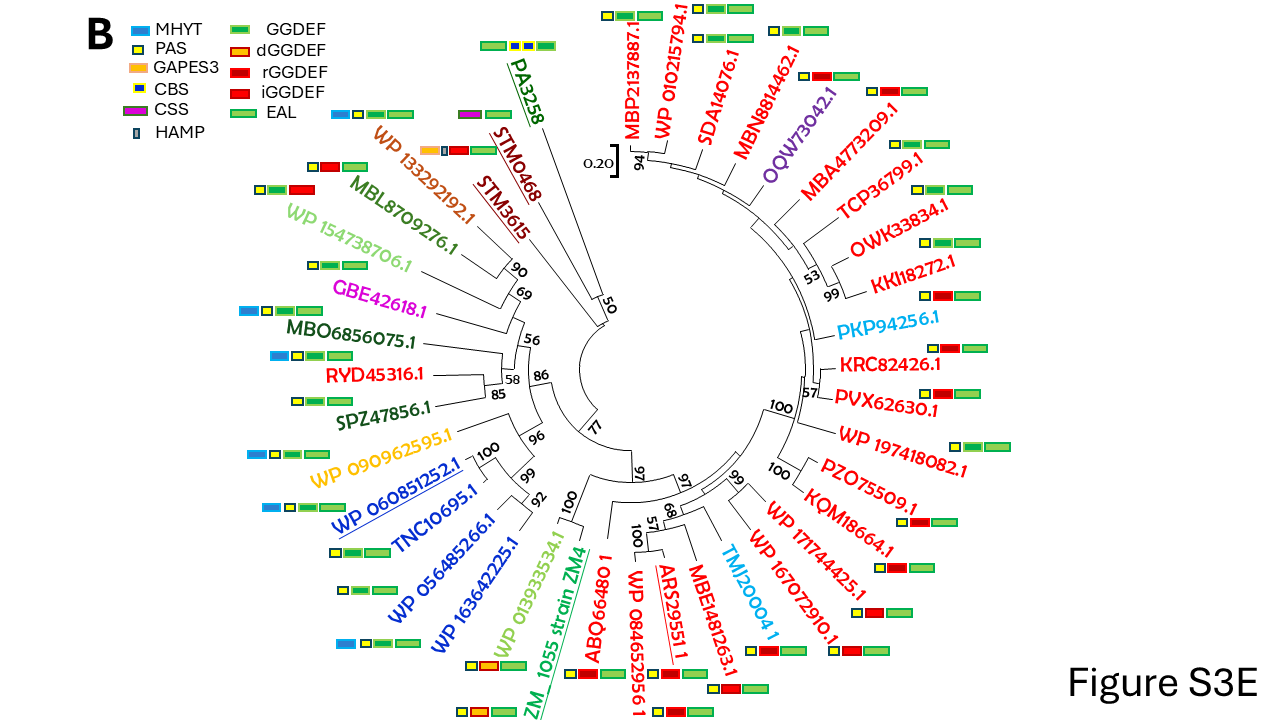


Figure S3b: Phylogenetic tree of the EAL domain from most similar homologous GGDEF-EAL proteins to the ZMO1055_ZM4_ EAL domain as retrieved by Blast (April 2021) from the NCBI database (Altschul et al. 1990) and the proteins used to create the A526V equivalent. The EAL domains of representatives of most similar PAS-GGDEF-EAL domain proteins have been aligned (Higgins and Sharp 1988) with subsequent manual curation and a Maximum-Likelyhood phylogenetic tree with 1000 bootstraps has been constructed in MEGA 7.0 (Kumar, Stecher and Tamura 2016). Next to the protein designation, the domain structure of the respective protein is given. Proteins used for mutational analysis are underlined. Symbols in the upper left corner indicate the different signaling and catalytic domains. GGDEF and EAL are predicted catalytically active diguanylate cyclase and phosphodiesterase domains. dGGDEF indicates degenerated GGDEF motif, but catalytically active domain as experimentally demonstrated. rGGDEF indicates predicted restricted catalytic activity with AGDEF/SGDEF motif, while iGGDEF indicates degenerated GGDEF (such as AADEF) including additional signature motif with no experimental documentation of catalytic activity. MHYT, PAS (Per-ARNT-Sim), GRAPE3, CBS (cystathionine-beta-synthase), CSS and HAMP (present in Histidine kinases, Adenyl cyclases, Methyl-accepting proteins and Phosphatases) are N-terminal signaling domains.


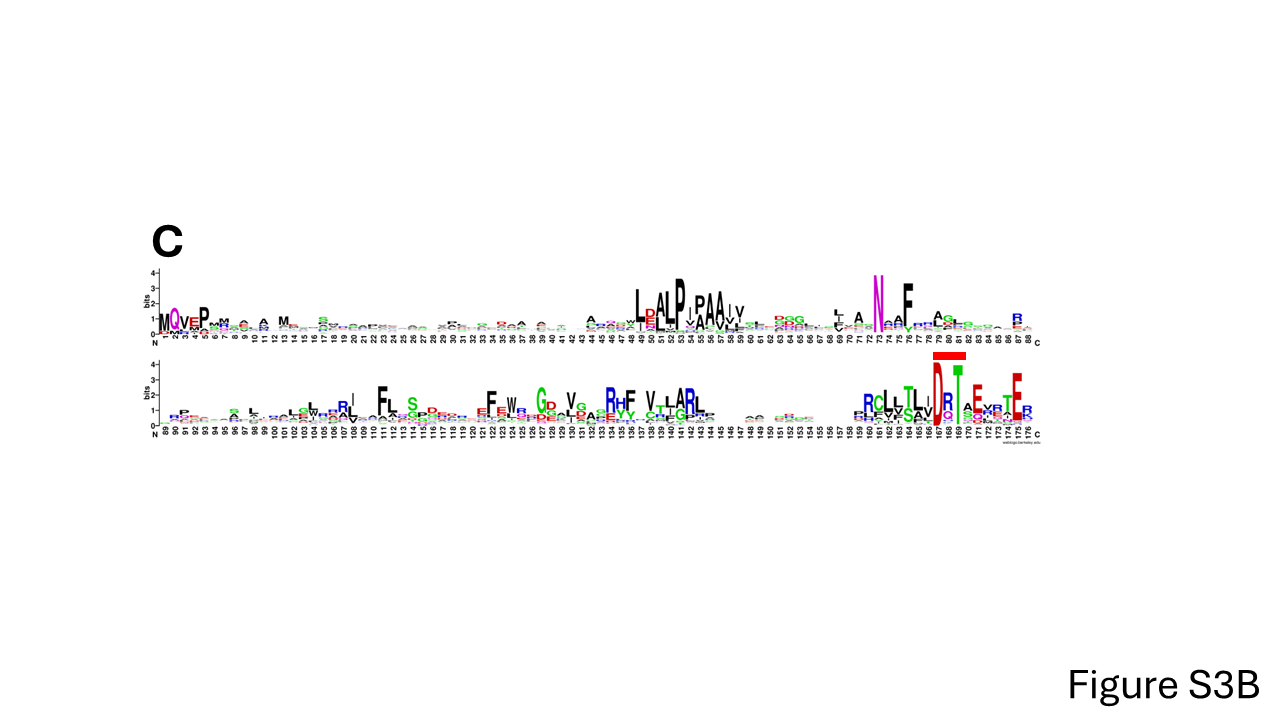


Figure S3c: Sequence logo of the ZMO1055_ZM4_ clade PAS domain. The PAS domain of the 100 most similar PAS-GGDEF-EAL domain proteins as retrieved by Blast (April 2021) (Altschul et al. 1990) has been aligned with Clustal 2.1 (Higgins and Sharp 1988) and a WebLogo (Crooks et al. 2004) has been created. Indicated by a red bar is the C-terminal end of the PAS domain (DxT motif).


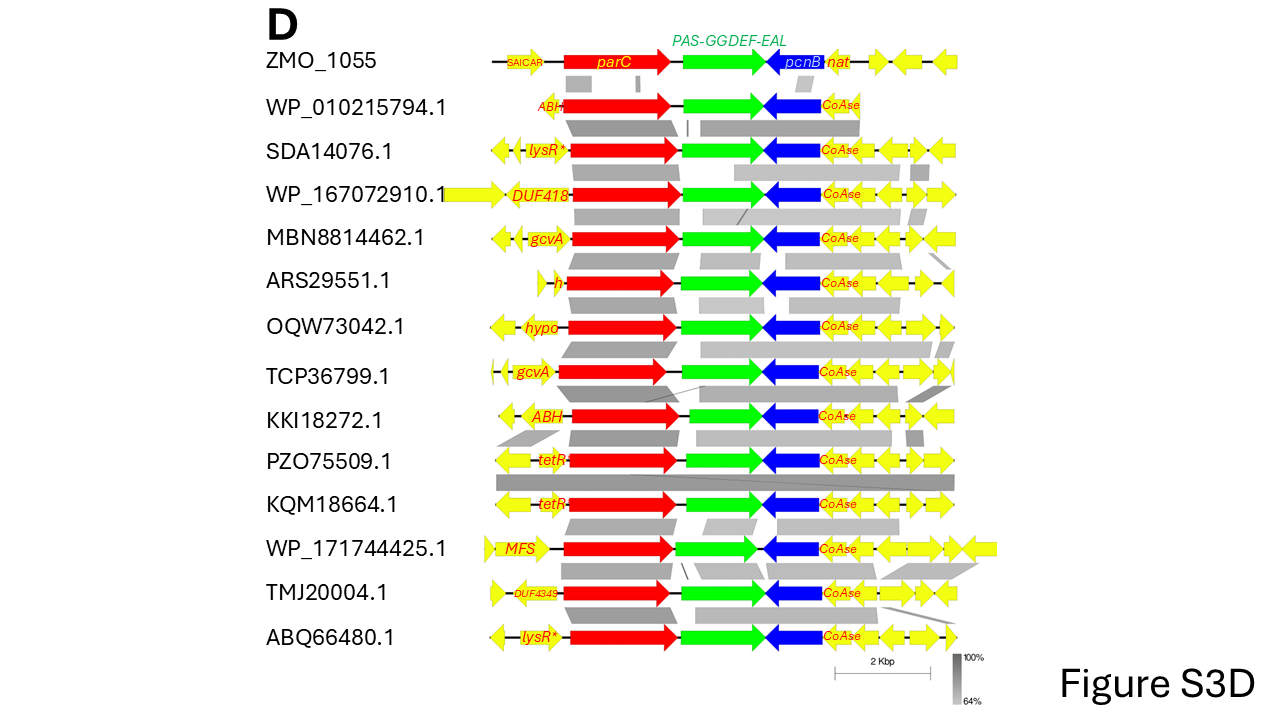


Figure S3d: Chromosomal position of the ZMO1055_ZM4_ homologs from the genus *Sphingomonas*. The immediate chromosomal position of most similar homologs of ZMO1055_ZM4_ from genus *Sphingomonas* representatives (with reference to the phylogenetic tree in Figure S3e) as displayed by Easyfig (Sullivan, Petty and Beatson 2011).


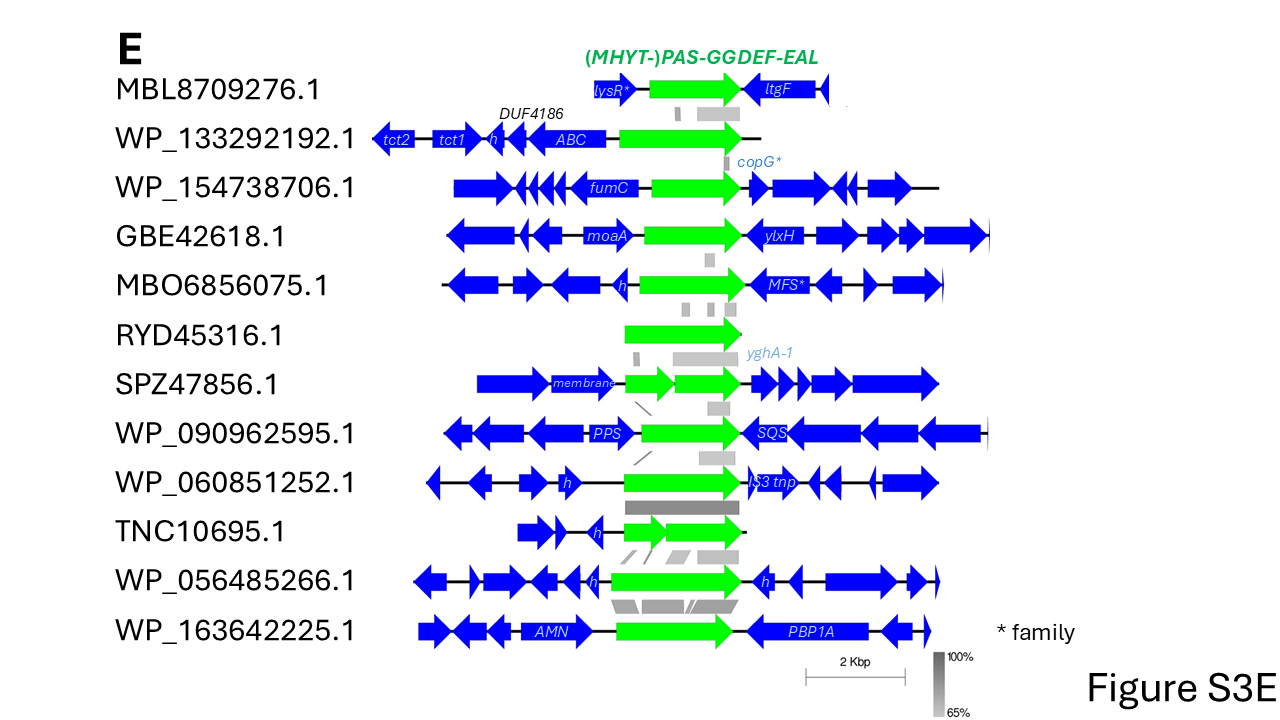


Figure S3e: Chromosomal position of the ZMO1055_ZM4_ homologs outside the genus *Sphingomonas*. The immediate chromosomal position of most similar homologs of ZMO1055_ZM4_ outside the genus *Sphingomonas* (with reference to the phylogenetic tree in Figure S3e) as displayed by Easyfig (Sullivan et al. 2011).


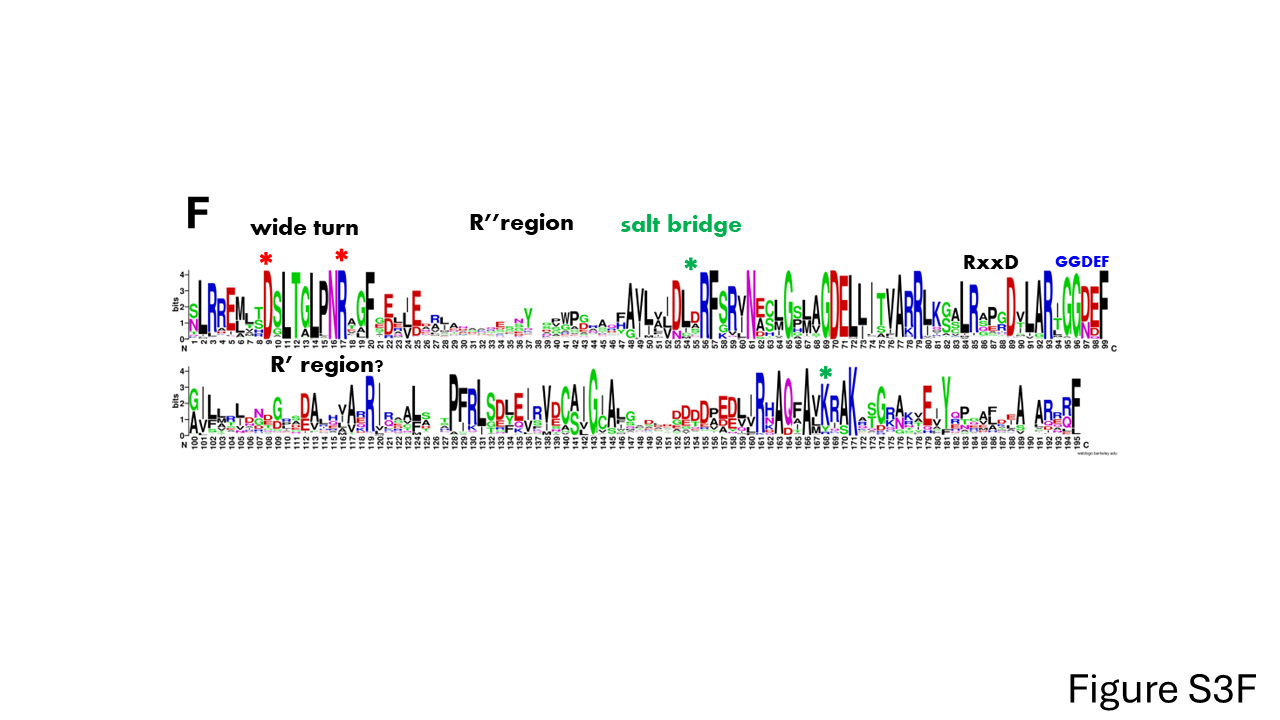


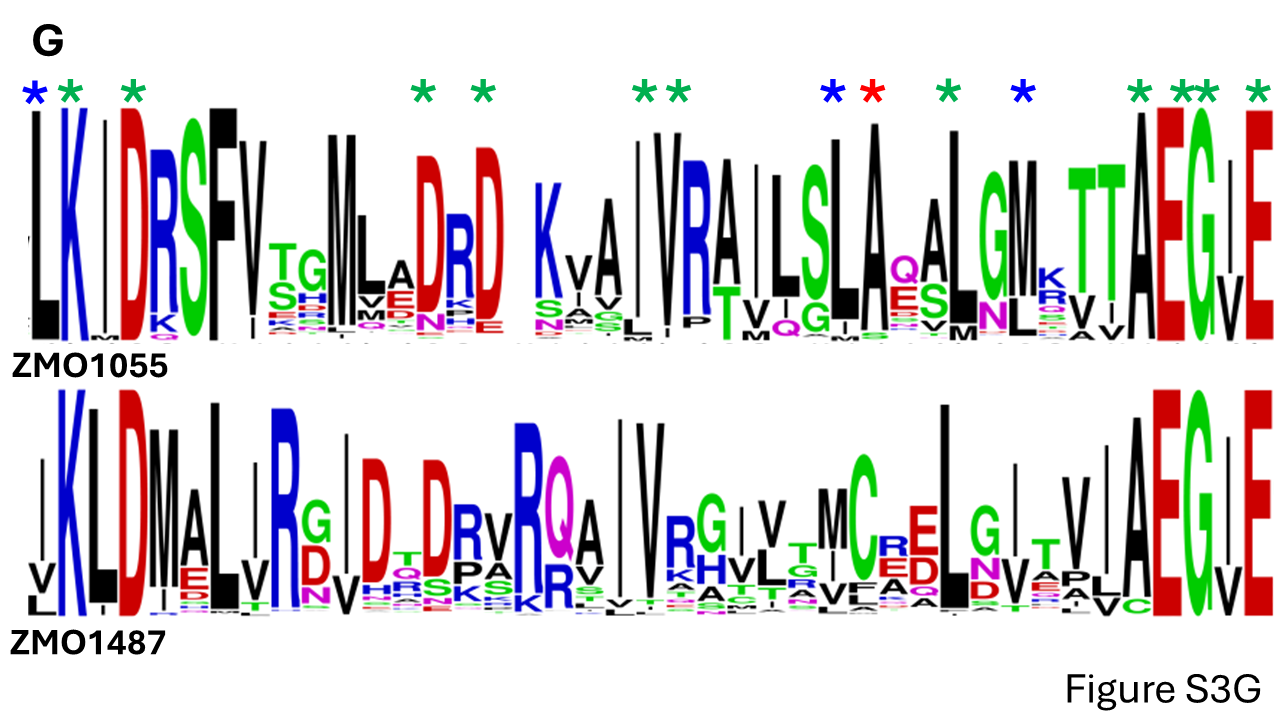


Figure S3f: Sequence logo (WebLogo) of the ZMO1055_ZM4_ clade GGDEF domains. The GGDEF domains from the most similar homologous GGDEF-EAL proteins to ZMO1055_ZM4_ as retrieved by Blast (April 2021) from the NCBI database (Altschul et al. 1990). The GGDEF domain of the 1000 most similar (MHYT)-PAS-GGDEF-EAL domain proteins as retrieved by Blast has been aligned (Higgins and Sharp 1988) with subsequent manual curation and a sequence logo has been constructed (Altschul et al. 1990). Functionally relevant motifs such as the catalytic site GGDEF motif, the allosteric I-site RxsD motif, the wide turn, the R’ and R’’ region, the wide turn and the salt bridge are indicated.

Figure S3g: Sequence logo of the amino acid sequence between the conserved KID and EGxE motif of ZMO1055_ZM4_ and ZMO1487_ZM4_ clade EAL domains. One thousand EAL domains of the most similar homologous GGDEF-EAL proteins to the ZMO1055_ZM4_ GGDEF domain and 1000 EAL domains of the most similar homologous proteins to ZMO1487_ZM4_ have been retrieved by Blast (October 2021) from the NCBI database (Altschul et al. 1990). The EAL domains were aligned with subsequent manual curation and a sequence logo has been retrieved (Crooks et al. 2004) displaying the amino acid sequence conservation between the conserved KID and EGxE motifs. Red star, position A526 in ZMO1055; blue stars, amino acids mutated in this study; green stars, reference amino acids highly conserved between ZMO1055 and ZMO1487 closest homologues.

**
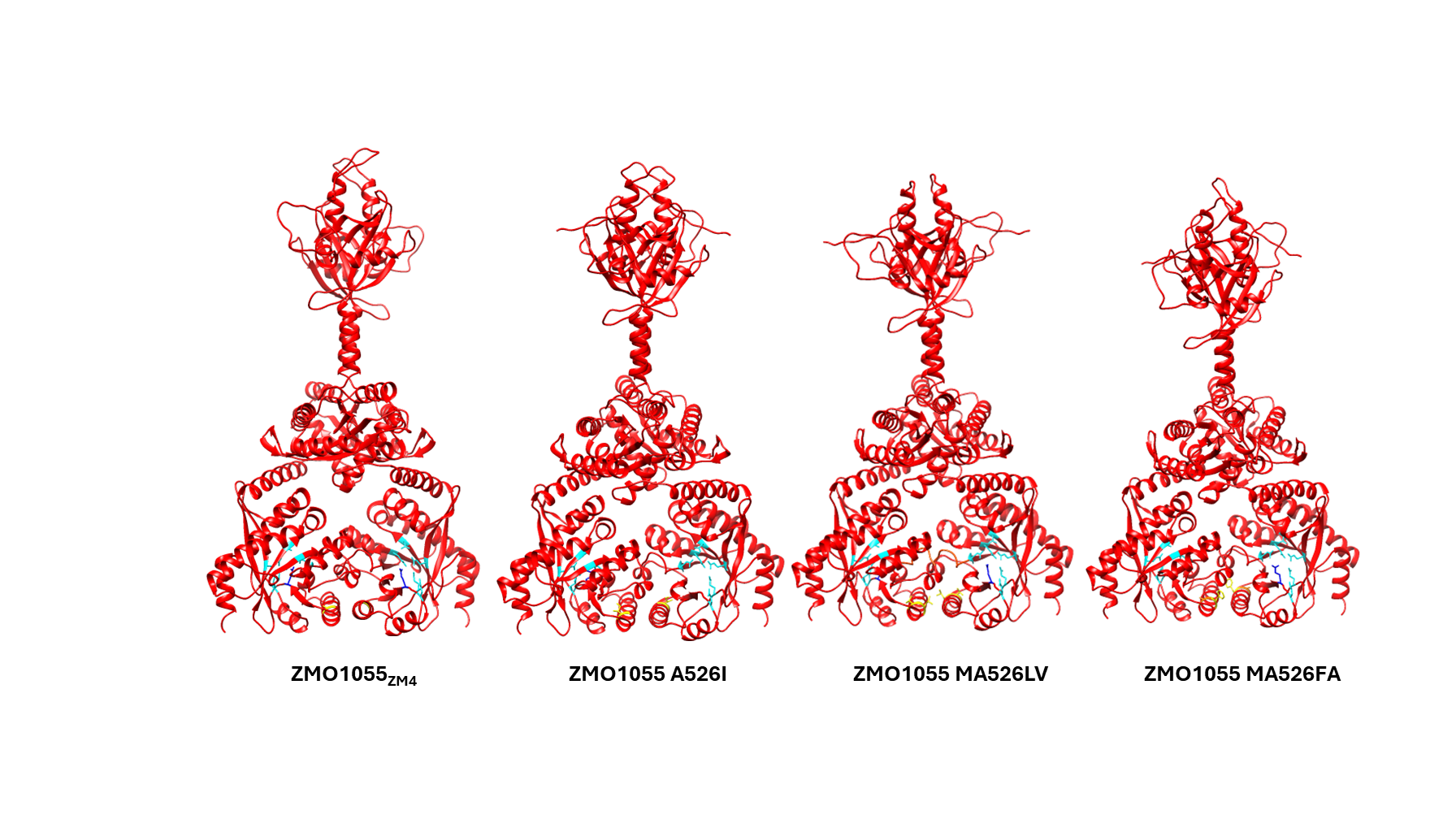
**

Figure S4: AlphaFold 3 model of the ZMO1055 dimer and selected variants. Yellow, substituted amino acids; light blue, catalytic site amino acids involved in divalent ion binding and, dark blue, catalytic base.

**References**

Altschul, S. F., W. Gish, W. Miller, E. W. Myers & D. J. Lipman (1990) Basic local alignment search tool. *J Mol Biol,* 215**,** 403-10.

Crooks, G. E., G. Hon, J. M. Chandonia & S. E. Brenner (2004) WebLogo: a sequence logo generator. *Genome Res,* 14**,** 1188-90.

Higgins, D. G. & P. M. Sharp (1988) CLUSTAL: a package for performing multiple sequence alignment on a microcomputer. *Gene,* 73**,** 237-44.

Kumar, S., G. Stecher & K. Tamura (2016) MEGA7: Molecular Evolutionary Genetics Analysis Version 7.0 for Bigger Datasets. *Mol Biol Evol,* 33**,** 1870-4.

Robert, X. & P. Gouet (2014) Deciphering key features in protein structures with the new ENDscript server. *Nucleic Acids Res,* 42**,** W320-4.

Sullivan, M. J., N. K. Petty & S. A. Beatson (2011) Easyfig: a genome comparison visualizer. *Bioinformatics,* 27**,** 1009-10.
