# Supplementary material for: Previously Uncharacterised Aliphatic Amino Acid Positions Modulate the Apparent Catalytic Activity of the EAL Domain of ZMO_1055 and Other Cyclic Di‐GMP‐Specific EAL Phosphodiesterases
Source: Microb Biotechnol. 2026 Feb 12;19(2):e70308. doi: 10.1111/1751-7915.70308 (PMC12900916; doi:10.1111/1751-7915.70308)
Supplement: Supplementary file 2 — Data S2: mbt270308‐sup‐0002‐Tables S1‐S3.docx. [file MBT2-19-e70308-s002.docx]

**Table S1. Strains constructed and used in this study**

**Strain designation Genotype Reference**

**in *E. coli* TOP10**

pEZ pEZ plasmid backbone from ﻿pEZ15a-Dual reporter This study

with deletion of sfGFP- and mCherry-related fragments

pEZ-1055 ZMO1055 from *Z. mobilis* ZM4 with 6×His-tag in the This study

C-terminal in pEZ plasmid

pEZ-1055(R221A) R221A mutation of ZM01055 This study

pEZ-1055(R228A) R228A mutation of ZM01055 This study

pEZ-1055(E356A) E356A mutation of ZM01055 This study

pEZ-1055(E536A) E536A mutation of ZM01055 This study

pEZ-EAL EAL domain of ZM01055 **(**305-579) This study

pEZ-PAS-GGDEF PAS-GGDEF domain of ZMO1055 (1-320) This study

pEZ-GGDEF GGDEF domain of ZMO1055 (139-320) This study

pEZ-1055_401 ZMO1055 from *Z. mobilis* ZM401 with 6×His-tag in the This study

C-terminal in pEZ plasmid

pEZ-1055(A526G) A526G mutation of ZM01055 This study

pEZ-1055(A526S) A526S mutation of ZM01055 This study

pEZ-1055(A526T) A526T mutation of ZM01055 This study

pEZ-1055(A526I) A526I mutation of ZM01055 This study

pEZ-1055(A526L) A526L mutation of ZM01055 This study

pEZ-1055(L499A) L499A mutation of ZM01055_401 This study

pEZ-1055(L499G) L499G mutation of ZM01055_401 This study

pEZ-1055(L499I) L499I mutation of ZM01055_401 This study

pEZ-1055(I531A) I531A mutation of ZM01055_401 This study

pEZ-1055(M525L) M525L mutation of ZM01055 This study

pEZ-1055(A526V, M525L) M525L mutation of ZM01055_401 This study

pEZ-1055(M525F) M525F mutation of ZM01055 This study

pEZ-ARS29551.1 ARS29551.1 contains GGDEF-EAL from *Sphingomonas* sp. This study

KC8, 6×His-tag in the C-terminus

pEZ-ARS29551.1(A521V) A526 homologous site mutation of ARS29551.1 This study

pEZ-ARS29551.1(E351A) PDE activity mutation (EAL > AAL) of ARS29551.1 This study

pEZ-WP_060851252.1(D466A) WP_060851252.1: MHYT-PAS-GGDEF-EAL with a DGC This study

GGDEF > GGAEF mutation of WP_060851252.1 from

*Methylobacterium aquaticum* MA-22A, 6×His-tag at the

C-terminal end

pEZ-WP_060851252.1 A526 homologous site mutation of WP_060851252.1(D466A) This study

(D466A,G733V)

pEZ-WP_060851252.1 PDE EAL > AAL mutation of WP_060851252.1(D466A) This study

(D466A,E569A)

pEZ-PA3258 PA3258 contains EAL-CBS-GGDEF from *Pseudomonas* This study

*aeruuginosa* SG17M, 6×His-tag at the C-terminal end

pEZ-PA3258(A221V) A526 homologous site mutation of PA3258 This study

pEZ-PA3258(E57A) PDE EAL > AAL mutation of PA3258 This study

pEZ-PA3258(wo his-tag) PA3258 contains EAL-CBS-GGDEF from *Pseudomonas* This study

*aerginosa* SG17M without 6xHis-tag

pEZ-PA3258(A221V,wo his-tag) A526 homologous site mutation of PA3258 without 6xHis-tag This study

pEZ-PA3258(E57A, wo his-tag) PDE EAL > AAL mutation of PA3258 without 6xHis-tag This study

pBAD30 pBAD plasmid backbone (1)

pBAD30-STM0468 YlaB with EAL domain from *S. typhimurium* UMR1 (ATCC (2)

14028 Nal^r^, rdar_28°C_), 8×His-tag at the C-terminal end

pBAD30-STM0468(A465V) A526 homologous site mutation of STM0468 This study

pBAD30-STM3615 GGDEF-EAL protein YhjK (STM3615) from *S. typhimurium* UMR1 (2, 3)

(ATCC 14028 Nal^r^, rdar_28°C_)

pBAD30-STM3615(A600V) A526 homologous site mutation of STM3615 This study

**in strain *Z. mobilis* ZM401**

pEZ *Z. mobilis* ZM401 with pEZ plasmid This study

pEZ-1055 *Z. mobilis* ZM401 with ZMO1055_ZM4_ in pEZ This study

pEZ-1055(R221A) *Z. mobilis* ZM401 with ZMO1055_ZM4_ R221A in pEZ This study

pEZ-1055(R228A) *Z. mobilis* ZM401 with ZMO1055_ZM4_ R228A in pEZ This study

pEZ-1055(E356A) *Z. mobilis* ZM401 with ZMO1055_ZM4_ E356A in pEZ This study

pEZ-1055(E536A) *Z. mobilis* ZM401 with ZMO1055_ZM4_ E536A in pEZ This study

pEZ-EAL *Z. mobilis* ZM401 with ZMO1055_ZM4_-EAL in pEZ This study

pEZ-PAS-GGDEF *Z. mobilis* ZM401 with ZMO1055_ZM4_-PAS-GGDEF in pEZ This study

pEZ-GGDEF *Z. mobilis* ZM401 with ZMO1055_ZM4_-GGDEF in pEZ This study

pEZ-1055_401 *Z. mobilis* ZM401 with ZMO1055_ZM401_ in pEZ This study

pEZ-1055(A526G) *Z. mobilis* ZM401 with ZMO1055_ZM4_ A526G in pEZ This study

pEZ-1055(A526S) *Z. mobilis* ZM401 with ZMO1055_ZM4_ A526S in pEZ This study

pEZ-1055(A526T) *Z. mobilis* ZM401 with ZMO1055_ZM4_ A526T in pEZ This study

pEZ-1055(A526I) *Z.* mobilis ZM401 with ZMO1055_ZM4_ A526I in pEZ This study

pEZ-1055(A526L) *Z. mobilis* ZM401 with ZMO1055_ZM4_ A526L in pEZ This study

pEZ-1055(L499A)_401 *Z. mobilis* ZM401 with ZMO1055_ZM401_ L499A in pEZ This study

pEZ-1055(L499G)_401 *Z. mobilis* ZM401 with ZMO1055_ZM401_ L499G in pEZ This study

pEZ-1055(L499I)_401 *Z. mobilis* ZM401 with ZMO1055_ZM401_ L499I in pEZ This study

pEZ-1055(I531A)_401 *Z. mobilis* ZM401 with ZMO1055_ZM401_ I531A in pEZ This study

pEZ-1055(M525L) *Z. mobilis* ZM401 with ZMO1055_ZM4_ M525L in pEZ This study

pEZ-1055(M525L)_401 *Z. mobilis* ZM401 with ZMO1055_ZM401_ M525L in pEZ This study

pEZ-1055(M525F) *Z. mobilis* ZM401 with ZMO1055_ZM4_ M525F in pEZ This study

in *S. typhimurium* UMR1 Δ*yhjH*

pEZ *S. typhimurium* UMR1Δ*yhjH* with pEZ plasmid This study

pEZ-ZMO1055 *S. typhimurium* UMR1 *ΔyhjH* with ZMO1055_ZM4_ in pEZ This study

pEZ-ZMO1055(R221A)_ZM4 *S. typhimurium* UMR1 Δ*yhjH* with R221A ZMO1055_ZM4_ in pEZ This study

pEZ-ZMO1055(R228A)_ZM4 *S. typhimurium* UMR1 Δ*yhjH* with R228A ZMO1055_ZM4_ in pEZ This study

pEZ-ZMO1055(E356A)_ZM4 *S. typhimurium* UMR1 Δ*yhjH* with E356A ZMO1055_ZM4_ in pEZ This study

pEZ-ZMO1055(E536A)_ZM4 *S. typhimurium UMR1 ΔyhjH* with E536A ZMO1055_ZM4_ in pEZ This study

pEZ-EAL_ZM4 *S. typhimurium* UMR1 Δ*yhjH* with ZMO1055_ZM4_-EAL in pEZ This study

pEZ-PAS-GGDEF *S. typhimurium* UMR1 Δ*yhjH* with ZMO1055_ZM4_-PAS-GGDEF This study

in pEZ

pEZ-GGDEF *S. typhimurium* UMR1 Δ*yhjH* with ZMO1055_ZM4_-GGDEF in pEZ This study

pEZ-ZMO1055_ZM401_ *S. typhimurium* UMR1 Δ*yhjH* with ZMO1055_ZM401_ in pEZ This study

pEZ-ZMO1055(A526G) *S. typhimurium* UMR1 Δ*yhjH* with A526G ZMO1055_ZM4_ in pEZ This study

pEZ-ZMO1055(A526S) *S. typhimurium* UMR1 Δ*yhjH* with A526S ZMO1055_ZM4_ in pEZ This study

pEZ-ZMO1055(A526T) *S. typhimurium UMR1* Δ*yhjH* with A526T ZMO1055_ZM4_ in pEZ This study

pEZ-ZMO1055(A526I) *S. typhimurium* UMR1Δ*yhjH* with A526I ZMO1055_ZM4_ in pEZ This study

pEZ-ZMO1055(A526L) *S. typhimurium* UMR1 Δ*yhjH* with A526L ZMO1055_ZM4_ in pEZ This study

pEZ-ZMO1055(L499A)_ZM401 *S. typhimurium* UMR1 Δ*yhjH* with L499A ZMO1055_ZM401_ in pEZ This study

pEZ-ZMO1055(L499G)_ZM401 *S. typhimurium* UMR1 Δ*yhjH* with L499G ZMO1055_ZM401_ in pEZ This study

pEZ-ZMO1055(L499I)_ZM401 *S. typhimurium* UMR1 Δ*yhjH* with ZMO1055_ZM401_ L499I in pEZ This study

pEZ-ZMO1055(l531A)_ZM401 *S. typhimurium* UMR1 Δ*yhjH* with ZMO1055_ZM401_ I531A in pEZ This study

pEZ-ZMO1055(M525L)_ZM4 *S. typhimurium* UMR1 Δ*yhjH* with ZMO1055_ZM4_ M525L in pEZ This study

pEZ-ZMO1055(M525L)_ZM401 *S. typhimurium* UMR1 Δ*yhjH* with M525L ZMO1055_ZM401_ in pEZ This study

pEZ-ZMO1055(M525F) *S. typhimurium* UMR1 Δ*yhjH* with ZMO1055_ZM4_ M525F in pEZ This study

pEZ-ARS29551.1 *S. typhimurium* UMR1 Δ*yhjH w*ith ARS29551.1 in pEZ This study

pEZ-ARS29551.1(A521V) *S. typhimurium* UMR1 Δ*yhjH* with ARS29551.1 A521V in pEZ This study

pEZ-ARS29551.1(E351A) *S. typhimurium* UMR1 Δ*yhjH* with ARS29551.1 E351A in pEZ This study

pEZ-WP_060851252.1(D446A) *S. typhimurium* UMR1 Δ*yhjH* with WP_060851252.1 D446A in pEZ This study

pEZ-WP_060851252.1 *S. typhimurium* UMR1 Δ*yhjH* with D446A and G733V This study

(D446A, G733V) WP_060851252.1 in pEZ

pEZ-WP_060851252.1 *S. typhimurium* UMR1 Δ*yhjH* with D446A and E569A This study

(D446A, E569A) WP_060851252.1 in pEZ

pEZ-PA3258 *S. typhimurium* UMR1 Δ*yhjH* with PA3258 in pEZ This study

pEZ-PA3258(A221V) *S. typhimurium* UMR1 Δ*yhjH* with PA3258 A221V in pEZ This study

pEZ-PA3258(E57A) *S. typhimurium* UMR1 Δ*yhjH* with PA3258 E57A ain pEZ This study

pEZ-PA3258(wo 6xHis-tag) *S. typhimurium* UMR1 Δ*yhjH* with PA3258 without C-terminal This study

6xHis-tag in pEZ

pEZ-PA3258(A221V, *S. typhimurium* UMR1 Δ*yhjH* with A221V PA3258 without This study

wo 6xHis-tag) C-terminal 6xHis-tag in pEZ

pEZ-PA3258(E57A, wo 6xHis-tag) *S. typhimurium* UMR1 Δ*yhjH* with E57A mutated PA3258 This study

without C-terminal 6xHis-tag in pEZ

pBAD30 *S. typhimurium* UMR1 Δ*yhjH* with pBAD30 plasmid This study

pBAD30-STM0468 *S. typhimurium* UMR1 Δ*yhjH* with STM0468 in pBAD30 This study

pBAD30-STM0468(A465V) *S. typhimurium* UMR1 Δ*yhjH* with STM0468 A465V in pBAD30 This study

pBAD30-STM3615 *S. typhimurium* UMR1 Δ*yhjH* with STM3615 in pBAD30 This study

pBAD30-STM3615(A600V) *S. typhimurium* UMR1 Δ*yhjH* with STM3615 A600V in pBAD30 This study

**Table S2. Plasmids constructed and used in this study**

**Plasmid Genotype Reference**

pEZ pEZ plasmid backbone from ﻿pEZ15a-Dual (4)

reporter with deletion of sfGFP- and mCherry-

related fragments

pEZ-1055 ZMO1055 from *Z. mobilis* ZM4 with 6×His-tag This study

at the C-terminus in pEZ

pEZ-1055(R221A) ZM01055 R221A in pEZ This study

pEZ-1055(R228A) ZM01055 R228A in pEZ This study

pEZ-1055(E356A) ZM01055 E356A in pEZ This study

pEZ-1055(E536A) ZM01055 E536A in pEZ This study

pEZ-EAL EAL domain (aa 305-579) of ZM01055 in pEZ This study

pEZ-PAS-GGDEF PAS-GGDEF domain (aa 1-320) of ZMO1055 This study

in pEZ

pEZ-GGDEF GGDEF domain (aa 139-320) of ZMO1055 in This study

pEZ

pEZ-1055_401 ZMO1055 from *Zymomonas mobilis* ZM401 This study

with a C-terminal 6×His-tag in pEZ

pEZ-1055(A526G) ZM01055 A526G in pEZ This study

pEZ-1055(A526S) ZM01055 A526S in pEZ This study

pEZ-1055(A526T) ZM01055 A526T in pEZ This study

pEZ-1055(A526I) ZM01055 A526I in pEZ This study

pEZ-1055(A526L) ZM01055 A526L in pEZ This study

pEZ-1055(L499A) ZM01055_401 L499A in pEZ This study

pEZ-1055(L499G) ZM01055_401 L499G in pEZ This study

pEZ-1055(L499I) ZM01055_401 L499I in pEZ This study

pEZ-1055(I531A) ZM01055_401 I531A in pEZ This study

pEZ-1055(M525L) ZM01055 M525L in pEZ This study

pEZ-1055(A526V, M525L) ZM01055_401 M525L in pEZ This study

pEZ-1055(M525F) ZM01055 M525F in pEZ This study

pEZ-ARS29551.1 ARS29551.1 GGDEF-EAL protein from This study

*Sphingomonas* sp., with C-terminal 6×His-tag in

pEZ

pEZ-ARS29551.1(A521V) ARS29551.1 with A526V equivalent substitution This study

in pEZ

pEZ-ARS29551.1(E351A) ARS29551.1 with E_351_AL > AAL mutation in This study

the EAL domain in pEZ

pEZ-WP_060851252.1 WP_060851252.1: MHYT-PAS-GGDEF-EAL This study

with C-terminal 6xHis-tag from *Methylo-*

*bacterium aquaticum* MA-22A in pEZ

pEZ-WP_060851252.1 WP_060851252.1 GGDEF > GGAEF catalytic This study

(D466A) mutant in pEZ

pEZ-WP_060851252.1 WP_060851252.1(D466A) with A526V This study

(D466A,G733V) equivalent substitution in pEZ

pEZ-WP_060851252.1 WP_060851252.1(D466A) with E_569_AL > AAL This study

(D466A,E569A) catalytic mutant in EAL domain in pEZ

pEZ-PA3258 PA3258: EAL-CBS-GGDEF from *Pseudomonas* This study

*aeruginosa* SG17M with C-terminal 6×His-tag

pEZ-PA3258(A221V) PA3258 with A526V equivalent substitution in This study

pEZ

pEZ-PA3258(E57A) PA3258 with E_57_AL > AAL catalytic mutant in This study

EAL domain in pEZ

pEZ-PA3258(wo 6xHis-tag) PA3258 in pEZ This study

pEZ-PA3258(A221V, PA3258 with A526V equivalent substitution in This study

wo 6xHis-tag) pEZ without 6xHis-tag

pEZ-PA3258(E57A, PA3258 with E_57_AL > AAL catalytic mutant in This study

wo 6xHis-tag) EAL domain without 6xHis-tag

pBAD30 pBAD plasmid backbone (1)

pBAD30-STM0468 YlaB with EAL domain from *S. typhimurium* (2)

UMR1 (ATCC 14028 Nal^r^, rdar_28°C_), 8×His-tag

at the C-terminal end

pBAD30-STM0468 STM0468 with A526V equivalent substitution in This study

(A465V) pBAD30

pBAD30-STM3615 YhjK with EAL domain from *S. typhimurium* (2, 3)

UMR1 (ATCC 14028 Nalr, rdar28°C)

pBAD30-STM3615 STM3615 with A526V equivalent substitution in This study

(A600V) pBAD30

**Table S3. Primers used in this study**

**primer name sequence description**

vector-his-tag-f GGCTACGGTCTCCatCACCACCACCACCACC Amplifying pEZ plasmid backbone with

ACtaaggatccaaactcgagtaag 6xHis-tag from ﻿pEZ15a-Dual reporter plasmid

vector-r GGCTACGGTCTCATCTTaactagcggagtgtacgg Amplifying pEZ plasmid backbone with

6xHis-tag from ﻿pEZ15a-Dual reporter

plasmid

promoter-f GGCTACGGTCTCAaagacccactttcacatttaag Amplifying *tetR*/*tetA* promoter from

from ﻿pEZ15a-Dual reporter plasmid

promoter-r GGCTACGGTCTCAtgggagatcctttctcctctttag Amplifying *tetR*/*tetA* promoter from

from ﻿pEZ15a-Dual reporter plasmid

1055-f GGCTACGGTCTCAcccatgccagatcctatatt Amplifying ZMO1055_ZM4_ or

aaaaac ZMO1055_ZM401_ with 6xHis-tag

1055-his-tag-r GGCTACGGTCTCCTAATTCCTGTTGGACGC Amplifying ZMO1055_ZM4_ or

CATATAG ZMO1055_ZM401_ with 6xHis-ta

within promoter-f cgacctcattaagcagctc locates in *tetR*/*tetA* promoter for

control Sanger sequencing

seq within 1055-f cctgcgtgacagtttgacg locates in ZMO1055 for control Sanger

sequencing

1055-walker-f cagcaatgggacaaacaagcc locates in ZMO1055 for control Sanger

sequencing

1055-R221A-f TTCAGTTTTAgccCCGGGAGATAGATTAG Forward primer for R221A mutation

GACGC

1055-R221A-r GTCAGACGCCGAGCAACT Reverse primer for R221A mutation

1055-R228A-f TAGATTAGGAgccATTGGCGGCGAC Forward primer for R228A mutation

1055-R228A-r TCTCCCGGCCTTAAAACT Reverse primer for R228A mutation

1055-E356A-f TGTTGGTTTCgccGCTTTAGCGC Forward primer for E356A mutation

1055-E356A-r ATTTTCTTATTATAGGGTGACAATATC Reverse primer for E356A mutation

1055-E536A-f AACGATTGTCgccGGCATCGAAAC Forward primer for E536A mutation

1055-E536A_4-r TTAATCGACAATGTCGAAG Reverse primer for E536A mutation

(pEZ)EAL-f ﻿ggatcatgtcgaagtctataatg Forward primer for constructing

ZMO1055 EAL domain pEZ

(pEZ)EAL-r atgggagatcctttctcctc Reverse primer for constructing

ZMO1055 EAL or GGDEF domain pEZ

(pEZ)PAS-GGDEF CCACCACCACCACCACtaa Forward primer for constructing

(his)-f ZMO1055 PAS-GGDEF domain pEZ

(pEZ)PAS-GGDEF ﻿TGtttttgtaagacgctggc Reverse primer for constructing PAS-(his)-r ZMO1055 PAS-GGDEF domain pEZ

GGDEF-f gACCCAAGCTGTCGAATTG Forward primer for constructing

ZMO1055 GGDEF domain pEZ

1055-A526G-f caccagcatgggttcgacattgtc Forward primer for A526G mutation

1055-A526S-f caccagcatgtcttcgacattgtc Forward primer for A526S mutation

1055-A526T-f caccagcatgacctcgacattgtcg Forward primer for A526T mutation

1055-A526I-f CACCAGCATGattTCGACATTGTCGATT Forward primer for A526I mutation

AAAAC

1055-A526L-f CACCAGCATGttgTCGACATTGTCGATT Forward primer for A526L mutation

AAAACGATTG

1055-A526G atagcgcgggtaatcgca Reverse primer for A526X mutation

/S/T/I/L-r

L499A-f ﻿tatcgatcatGCCaaaatagatcgcag Forward primer for L499A mutation

L499G-f tatcgatcatGGTaaaatagatcgcag Forward primer for L499G mutation

L499I-f tatcgatcatATTaaaatagatcgcag Forward primer for L499I mutation

L499A/G/I-r ﻿gggaaagttttcagataag Reverse primer for L499X mutation

I531A-f ﻿gacattgtcgGCCaaaacgattgtcg Forward primer for I531A mutation

I531A-r ﻿gaaaccatgctggtgatag Reverse primer for I531A mutation

1055_4-M525L-f tatcaccagcttggcttcgacattg Forward primer for M525L mutation of ZMO1055

1055_401-M525L-f tatcaccagcttggtttcgacattg Forward primer for M525L mutation

of ZMO1055_ZM401_

1055-M525L-r gcgcgggtaatcgcaatc Reverse primer for M525L mutation

ARS29551.1 XhoI-r ccgctcgagtttggatccTCAGTGGTGGTGGTGG Amplifying ARS29551.1 with 6xHis-tag TGGTGTGC flanked by restriction sites to be

cloned into pEZ

ARS29551.1 EcoRI-f ccggaattcATGGTCTCGGCGTTCGACAGCG Amplifying ARS29551.1 with 6xHis-tag

flanked by restriction sites to be

cloned into pEZ

pEZ EcoRI-r ﻿ccggaattcgggagatcctttctcctctttagatc Amplifying pEZ plasmid backbone with

6xHis-tag to ligate with ARS29551.1,

WP_060851252.1 or PA3258

pEZ XhoI-f ccgctcgagtaaggatctccaggc Amplifying pEZ plasmid backbone with

6xHis-tag to ligate with ARS29551.1,

WP_060851252.1 or PA3258

ARS29551.1 ﻿ACTGGGCCTTgttGACGCGTTAGGAATG Forward primer for A521V mutation

(A521V)-f

ARS29551.1 ﻿ACGGCCCGAACAATCGCT Reverse primer for A521V mutation

(A521V)-r

ARS29551.1 CGCAGGGTTTgccGCACTTGCTC Forward primer for E351A mutation

(E351A)-f

ARS29551.1 ACCTTGTTTGTTTGTAAGTTG Reverse primer for E351A mutation

(E351A)-r

ARS29551.1 ﻿CTTACAAACAAACAAGGTCGCA locates in ARS29551.1 for walker-f control Sanger sequencing

WP_060851252.1 ﻿ccggaattcATGCAGCTCCCGCTCACCCAC Amplifying WP_060851252.1 with protein EcoRI-f 6xHis-tag and flanked by restriction

sites to be ligated into pEZ

WP_060851252.1 ﻿ccgctcgagtttggatccttaGTGGTGGTGGTG Amplifying WP_060851252.1 with

protein XhoI GTGGTGATTGACATGT 6xHis-tag and flanked by restriction

(with his-tag)-r sites to be ligated into pEZ

WP_060851252.1 GCTTGGCGGTGCCGAGATGGCGG Forward primer for D446A mutation

(D446A)-f

WP_060851252.1 CGCGCCACAAAGCCCACTTC Reverse primer for D446A mutation

(D446A)-r

WP_060851252.1 CACGCAGATGgttAGCGACATGG Forward primer for G733V mutation

(G733V)-f

WP_060851252.1 ATCGCCTTGACAACTGCC Reverse primer for G733V mutation

(G733V)-r

WP_060851252.1 CGTCGGCTACgccGCGCTCATAC Forward primer for E569A mutation

(E569A)-f

WP_060851252.1 ATGGCGTCATTGGAGAGC Reverse primer for E569A mutation

(E569A)-r

WP_060851252.1 ﻿CTCGAAGGGCAAGAAACAATAC Sequence locates in WP_060851252.1 walker 0.5-f for control Sanger sequencing

h-PA3258-f ctaaagaggagaaaggatctcccgtgaaatacggg Amplifying PA3258 flanked by

gccggttgc homologous region to be recombined

into pEZ plasmid backbone

h-PA3258-r gatccttactcgagtttggatccttaggcggcctcga Amplifying PA3258 flanked by

tcaggtg homologous region to be recombined

into pEZ plasmid backbone

hpEZ(for PA3258)-f gatcgaggccgcctaaggatccaaactcgagtaa Amplifying pEZ plasmid backbone with ggatctc 6xHis-tag to be recombined with

PA3258

hpEZ(for PA3258)-r accggccccgtatttcacgggagatcctttctcctct Amplifying pEZ plasmid backbone with

ttagatc 6xHis-tag to be recombined with

PA3258

(PA3258)his-tag-f CCACCACtaaggatccaaactcgagtaaggatctcc Forward primer for adding 6×His-tag

At the C-terminal end of PA3258

(PA3258)his-tag-r tggtggtggtgGGCGGCCTCGATCAGGTG Reverse primer for adding 6×His-tag in

the C-terminal end of PA3258

PA3258- ctggaactactctgccgacaac Sequence locates in PA3258 for walker0.5-F control Sanger sequencing

PA3258(A221V)-f cctgaagatggttcgggcctctc Forward primer for A221V mutation

PA3258(A221V)-r ﻿atggagccgacgaactcg Reverse primer for A221V mutation

(PA3258)E57A-f ggtcggctacGCCgcgctgaccc Forward primer for E57A mutation

(PA3258)E57A-r aggcgccgttcggaaagc Reverse primer for E57A mutation

YlaB A465V-r aatatgcggcgtgacgtt Forward primer for A465V mutation

YlaB A465V-f attgaaatggtgaaaacgctcaaac Reverse primer for A465V mutation

YhjK A600V-r atcgccgtcaccatactg Forward primer for A600V mutation

YhjK A600V-f tatcctgatggtgcgcagtcttaatttac Reverse primer for A600V mutation

**References**

1. Guzman LM, Belin D, Carson MJ, Beckwith J. 1995. Tight regulation, modulation, and high-level expression by vectors containing the arabinose PBAD promoter. J Bacteriol 177:4121-30.

2. Simm R, Lusch A, Kader A, Andersson M, Romling U. 2007. Role of EAL-containing proteins in multicellular behavior of Salmonella enterica serovar Typhimurium. J Bacteriol 189:3613-23.

3. Anwar N, Rouf SF, Romling U, Rhen M. 2014. Modulation of biofilm-formation in Salmonella enterica serovar Typhimurium by the periplasmic DsbA/DsbB oxidoreductase system requires the GGDEF-EAL domain protein STM3615. PLoS One 9:e106095.

4. Yang Y, Rong Z, Song H, Yang X, Li M, Yang S. 2020. Identification and characterization of ethanol-inducible promoters of Zymomonas mobilis based on omics data and dual reporter-gene system. Biotechnol Appl Biochem 67:158-165.
